# Gain and loss of TASK3 channel function and its regulation by novel variation cause *KCNK9* imprinting syndrome

**DOI:** 10.1186/s13073-022-01064-4

**Published:** 2022-06-13

**Authors:** Margot A. Cousin, Emma L. Veale, Nikita R. Dsouza, Swarnendu Tripathi, Robyn G. Holden, Maria Arelin, Geoffrey Beek, Mir Reza Bekheirnia, Jasmin Beygo, Vikas Bhambhani, Martin Bialer, Stefania Bigoni, Cyrus Boelman, Jenny Carmichael, Thomas Courtin, Benjamin Cogne, Ivana Dabaj, Diane Doummar, Laura Fazilleau, Alessandra Ferlini, Ralitza H. Gavrilova, John M. Graham, Tobias B. Haack, Jane Juusola, Sarina G. Kant, Saima Kayani, Boris Keren, Petra Ketteler, Chiara Klöckner, Tamara T. Koopmann, Teresa M. Kruisselbrink, Alma Kuechler, Laëtitia Lambert, Xénia Latypova, Robert Roger Lebel, Magalie S. Leduc, Emanuela Leonardi, Andrea M. Lewis, Wendy Liew, Keren Machol, Samir Mardini, Kirsty McWalter, Cyril Mignot, Julie McLaughlin, Alessandra Murgia, Vinodh Narayanan, Caroline Nava, Sonja Neuser, Mathilde Nizon, Davide Ognibene, Joohyun Park, Konrad Platzer, Céline Poirsier, Maximilian Radtke, Keri Ramsey, Cassandra K. Runke, Maria J. Guillen Sacoto, Fernando Scaglia, Marwan Shinawi, Stephanie Spranger, Ee Shien Tan, John Taylor, Anne-Sophie Trentesaux, Filippo Vairo, Rebecca Willaert, Neda Zadeh, Raul Urrutia, Dusica Babovic-Vuksanovic, Michael T. Zimmermann, Alistair Mathie, Eric W. Klee

**Affiliations:** 1 Department of Quantitative Health Sciences, Mayo Clinic, Rochester, MN USA; 2 Center for Individualized Medicine, Mayo Clinic, Rochester, MN USA; 3 Medway School of Pharmacy, University of Kent and University of Greenwich, Central Avenue, Anson Building, Central Avenue, Chatham Maritime, ME4 4, Kent, TB ME4 4 TB UK; 4 Bioinformatics Research and Development Laboratory, Linda T. and John A. Mellowes Center for Genomic Sciences and Precision Medicine, Medical College of Wisconsin, Milwaukee, WI USA; 5 Department for Women and Child Health, Hospital for Children and Adolescents, University Hospitals, University of Leipzig, Leipzig, Germany; 6 Children’s Hospital of Minnesota, Minneapolis, MN USA; 7 Department of Molecular and Human Genetics, Baylor College of Medicine, Houston, TX USA; 8 Institute of Human Genetics, University Hospital Essen, University of Duisburg-Essen, Essen, Germany; 9 Division of Medical Genetics, Northwell Health, Manhasset, NY USA; 10 Medical Genetics Unit, Department of Medical Sciences, Ferrara University, Ferrara, Italy; 11 Division of Neurology, BC Children’s Hospital, Vancouver, British Columbia Canada; 12 Oxford Centre for Genomic Medicine, ACE Building, Nuffield Orthopaedic centre, Oxford University Hospitals NHS Foundation Trust, Windmill road, Headington, Oxford, OX3 7HE UK; 13 Département of Genetics, APHP, Hôpital Pitié-Salpêtrière, Sorbonne Université, Paris, France; 14 CHU Nantes, Service de génétique médicale, Nantes, France; 15 CHU de Rouen, Service de Néonatologie, Réanimation pédiatrique, Neuropédiatrie et éducation fonctionnelle de l’enfant, INSERM U 1245, ED497, 76000 Rouen, France; 16 APHP, Hôpital Raymond Poincaré, Hôpitaux Universitaires Paris Ile-de-France Ouest, Pôle pédiatrique, Service de Pédiatrie, Centre de Reference Nord-Est-Ile de France, 92380 Garches, France; 17 APHP, Department of Neuropediatrics, National Reference Center for Neurogenetic Disorders, Hôpital Armand-Trousseau, GHUEP, Paris, France; 18 Service de Néonatologie, CHU de Caen, Caen, France; 19 Department of Clinical Genomics, Mayo Clinic, Rochester, MN USA; 20 Department of Pediatrics, Harbor-UCLA Medical Center, Cedars-Sinai Medical Center, David Geffen School of Medicine at UCLA, Los Angeles, CA USA; 21 Centre for Rare Diseases, University of Tübingen, Tübingen, Germany; 22 Institute of Medical Genetics and Applied Genomics, University of Tübingen, Tübingen, Germany; 23 GeneDx, 207 Perry Parkway, Gaithersburg, MD USA; 24 Department of Clinical Genetics, Leiden University Medical Center, Leiden, The Netherlands; 25 Department of Clinical Genetics, Erasmus MC, University Medical Center Rotterdam, Rotterdam, The Netherlands; 26 Departments of Pediatrics and Neurology, University of Texas Southwestern Medical Center and Children’s Health, Dallas, TX USA; 27 APHP, Département de Génétique et Centre de Référence Déficiences Intellectuelles de Causes Rares, Hôpital de la Pitié-Salpêtrière, Assistance Publique - Hôpitaux de Paris, 75651 Paris, France; 28 Pediatrics III, Pediatric Oncology and Hematology, University Hospital Essen, Essen, Germany; 29 Institute of Human Genetics, University of Leipzig Medical Center, 04103 Leipzig, Germany; 30 Service de Genetique Clinique, CHRU de Nancy, F-54000 Vandoeuvre-les-Nancy, France; 31 Unite INSERM N-GERE UMR_S 1256, Université de Lorraine, Faculté de Médecine, 9 avenue de la Forêt de Haye, CS 50184, Vandoeuvre-les-Nancy, France; 32 Section of Medical Genetics, SUNY Upstate University Hospital, Syracuse, NY USA; 33 Molecular Genetics of Neurodevelopmental Disorders, Department of Woman and Child Health, University of Padova, Padua, Italy; 34 Pediatric Research Institute, Città della Speranza, Padova, Italy; 35 Department of Paediatric Medicine, KK Women’s and Children’s Hospital, Mount Elizabeth Hospital, Singapore, Singapore; 36 Texas Children’s Hospital, Houston, TX USA; 37 Division of Plastic and Reconstructive Surgery, Mayo Clinic, Rochester, MN USA; 38 Center for Rare Childhood Disorders, Translational Genomics Research Institute, Phoenix, AZ USA; 39 Department of Genetics, Reims University Hospital, Reims, France; 40 Joint BCM-CUHK Center of Medical Genetics, Shatin, Hong Kong SAR; 41 Department of Pediatrics, Division of Genetics and Genomic Medicine, Washington University School of Medicine, St. Louis, MT USA; 42 Practice of Human Genetics, Bremen, Germany; 43 Genetics Center, Orange, CA USA; 44 Division of Medical Genetics, CHOC Children’s Hospital, Orange, CA USA; 45 Department of Surgery, Medical College of Wisconsin, Milwaukee, WI USA; 46 Clinical and Translational Sciences Institute, Medical College of Wisconsin, Human Research Center, Milwaukee, Wl USA; 47 Department of Biochemistry, Medical College of Wisconsin, Milwaukee, WI USA; 48 School of Engineering, Arts, Science and Technology, University of Suffolk, Ipswich, UK

**Keywords:** *KCNK9* imprinting syndrome, TASK3 channel, Neurodevelopmental disorder, Electrophysiology, Computational protein modeling

## Abstract

**Background:**

Genomics enables individualized diagnosis and treatment, but large challenges remain to functionally interpret rare variants. To date, only one causative variant has been described for *KCNK9* imprinting syndrome (KIS). The genotypic and phenotypic spectrum of KIS has yet to be described and the precise mechanism of disease fully understood.

**Methods:**

This study discovers mechanisms underlying *KCNK9* imprinting syndrome (KIS) by describing 15 novel *KCNK9* alterations from 47 KIS-affected individuals. We use clinical genetics and computer-assisted facial phenotyping to describe the phenotypic spectrum of KIS. We then interrogate the functional effects of the variants in the encoded TASK3 channel using sequence-based analysis, 3D molecular mechanic and dynamic protein modeling, and in vitro electrophysiological and functional methodologies.

**Results:**

We describe the broader genetic and phenotypic variability for KIS in a cohort of individuals identifying an additional mutational hotspot at p.Arg131 and demonstrating the common features of this neurodevelopmental disorder to include motor and speech delay, intellectual disability, early feeding difficulties, muscular hypotonia, behavioral abnormalities, and dysmorphic features. The computational protein modeling and in vitro electrophysiological studies discover variability of the impact of *KCNK9* variants on TASK3 channel function identifying variants causing gain and others causing loss of conductance. The most consistent functional impact of *KCNK9* genetic variants, however, was altered channel regulation.

**Conclusions:**

This study extends our understanding of KIS mechanisms demonstrating its complex etiology including gain and loss of channel function and consistent loss of channel regulation. These data are rapidly applicable to diagnostic strategies, as KIS is not identifiable from clinical features alone and thus should be molecularly diagnosed. Furthermore, our data suggests unique therapeutic strategies may be needed to address the specific functional consequences of *KCNK9* variation on channel function and regulation.

**Supplementary Information:**

The online version contains supplementary material available at 10.1186/s13073-022-01064-4.

## Background

The field of clinical genomics is facing a challenge to the paradigm that single genetic variants define specific human diseases, or if a spectrum of genetic changes can produce the same disease, or even be therapeutically treated in the same way. Elucidating the precise mechanism of disease, especially for channelopathies where agonists or antagonists may be clinically available, may suggest specific pharmacotherapeutic interventional opportunities. *KCNK9* (MIM: 605874, NM_001282534.1) encodes the TASK3 (TWIK-related acid-sensitive K channel 3, K_2P_9.1) protein, a member of the two-pore domain potassium (K2P) channel family [1, 2]. *KCNK9* is among a small number of paternally imprinted genes where only the maternal allele is expressed [3]. Recent mouse studies, however, have detected residual paternal expression in some brain regions [4]. *KCNK9* imprinting syndrome (KIS), also known as Birk-Barel syndrome (MIM: 612292), is a rare genetic disorder caused by a genetic alteration of the maternal copy of *KCNK9,* first reported with the causal variant, p.(Gly236Arg) [5], and two subsequent variants of uncertain significance (VUS) [6, 7]. KIS is characterized by pathophysiological symptoms, of variable severity, including dysmorphic features with elongated face, varying degrees of intellectual disability (ID), and congenital hypotonia [5, 8].

K2P channels participate in the stabilization of the resting membrane potential of excitable and non-excitable cells, regulating cell activity. *KCNK9*-encoded TASK3 channels are predominantly expressed in the central nervous system (CNS) where they contribute to background current in many neuronal populations [9, 10]. TASK3 knockout mice show a number of cognitive impairments, and these channels are proposed to play a role in various pathologic conditions including epilepsy, pain and disorders of aldosterone secretion, respiratory stimulation, and sleep duration [9]. Highly regulated by physiological mediators, these channels are particularly sensitive to increased extracellular acidification, resulting in channel inhibition [1, 9]. These channels are also potently inhibited by activated G-protein-coupled receptors (GPCRs) that couple primarily through the G_αq_ protein family [11–13].

Because of the importance of this channel to human pathophysiology, several laboratories, including ours, have devoted efforts to mechanistically characterize its wild-type and variant-altered function. Electrophysiological studies of the p.Gly236Arg variant demonstrated reduced inwardly rectifying currents which is insensitive to extracellular pH- and GPCR-mediated regulation [14]. Transient expression of the p.Gly236Arg variant in cortical pyramidal neurons during development severely impaired migration, likely contributing to developmental disorder in KIS [15]. Mice lacking TASK3 (*Kcnk9*^-/-^) have impaired memory [16], sleep perturbation [17], and resistance to despair behavior that has a link to depression [18]. Assuming a loss-of-function mechanism, treatment strategies have been proposed using channel-stimulatory drugs [8]. However, the precise mechanisms of KIS, and whether additional variants cause the same disorder has remained unknown.

The current study significantly advances the knowledge of this syndrome by comprehensively assessing 47 individuals with 19 unique *KCNK9* variants. We characterize 15 novel variants using data modeling and cellular experiments to determine how each alteration changes channel function and interpret these data in the context of the clinical findings. We found that *KCNK9* variants can cause gain or loss of channel function, and both exhibit the pathognomonic characteristic of altered regulation. Importantly, gain-of-function alleles thus may not be amenable to channel-stimulatory drugs. Thus, this study provides new knowledge of significant biomedical relevance by advancing our current understanding of this syndrome and its pathobiological mechanisms while also demonstrating how computational and experimental methods can be integrated to solve disease mechanisms.

## Methods

### Participants

This study describes 47 affected individuals from 29 families. We describe 26 newly identified individuals from 22 families (families F1-17, F23, F24, F26, F27, and F29) accrued through professional communication as well as through GeneMatcher [19]. They were recruited from hospitals or clinics from the USA and other countries including the UK, Germany, Italy, France, The Netherlands, Canada, and Singapore. Clinical, phenotypic, and *KCNK9* genetic variant data were analyzed for each participant. All families providing new or updated data provided informed consent (F1-F19, F23, F24, F26-F27, and F29). Data for families F20-F22, F25 and F28 was abstracted from prior publications only. This study was approved by the Mayo Clinic Institutional Review Board, and local Ethics Committees. Family 9 was recruited to the Genomics England 100,000 Genomes Project. Previously published individuals with *KCNK9* variants are also described with summarized published data and/or updated unpublished clinical data [5–8]. Persons P18.1, P19.1, P20.1, and P21.1 in this study refer to the published Patients 1-4 from Graham et al. [8]. Family F22 refers to the Arab-Israeli kindred described by both Barel et al. and Graham et al. [5, 8]. Person P25.1 refers to the individual reported by Šedivá et al. [6], Family F27 was previously reported without detailed clinical information [20], and person P28.1 refers to BK-227-03 described by Guo et al. [7].

### Variant identification

*KCNK9* variants were identified through a variety of methods including gene panel testing or exome or genome sequencing through commercial clinical laboratories including Ambry Genetics (Aliso Viejo, CA), Baylor Genetics (Houston, TX), and GeneDx (Gaithersburg, MD), academic clinical laboratories, or through research studies. Additional details are provided in the individual clinical histories in the Additional file 1: Supplementary Note.

### Facial analysis

Face2Gene Research application (FDNA Inc., Boston, MA) using DeepGestalt technology (algorithm 19.1.9) [21] was used to evaluate the presence of a distinct facial pattern in individuals with *KCNK9* imprinting syndrome. Seventeen frontal photos of the face were obtained from unrelated affected individuals without glasses or black eye bars (individual: age at photo: P3.1: 9y, P6.1: 10y3 m, P7.1: 9y, P8.1: 1y5 m, P9.1: 12y-18y, P12.1: 2y2 m; P13.1: 6y1m, P16.1: 17y, P18.1 (updated photo patient 1) [8]: 6y, P23.1: 1y3 m, P24.1: 3 m, P27.1: 6y1m, P29.1: 8y1m and published photos of patient 2 (P19.1: 11 m), 3 (P20.1: 1y1m), and 4 (P21.1: 3y) from Graham et al. [8] and the individual (P25.1: 17y) described by Šedivá et al. [6] and compared to 17 control images matched for age, sex, and ethnicity provided by Face2Gene. To estimate the power of DeepGestalt in distinguishing affected individuals from controls, a cross validation scheme was used, including a series of binary comparisons between all groups. For these binary comparisons, the data was split randomly multiple times into training sets and test sets. Each such set contained half of the samples from the group, and this random process was repeated 10 times. The results of the comparisons are reported using the receiver operating characteristic (ROC) curve and area under the curve (AUC). The mean AUC = 0.87 and AUC STD = 0.07.

### Molecular modeling

No experimentally solved structure currently exists for the protein encoded by *KCNK9*, TASK3. Therefore, we began molecular modeling from the canonical UniProt sequence, Q9NPC2-1, which corresponds to Ensembl transcript ENST00000303015 to search for existing experimental structures of homologous sequences using Clustal [22, 23] alignment to the Protein Data Bank (PDB) [24]. Using I-tasser [25], we generated homology-based models for the highest homology experimental structures - residues 1-271 from human KCNK1 (PDB: 3ukm) [26] and KCNK4 (PDB: 3um7) [27]. These models were evaluated for quality using online servers and standard metrics including PROCHECK [28], QMEAN [29], QMEANBrane [30], and VADAR [31]. We compared models to one another by calculations of their electrostatic potentials, volumes, and accessible surface areas using DaliLite [32] and APBS [33]. We summarized quality metrics at the residue level to evaluate if differences in quality clustered in 3D. All structure metrics were within expectation for high-confidence membrane proteins. Regions that are embedded within the membrane have high quality scores, comparable to solvent-exposed loops on both intracellular and extracellular-facing sides, which have lower quality scores. We believe that these loops will be more flexible, and therefore, any individual static representation of them will be insufficient. Our use of replicates and comparison to wildtype (WT) will help control for errors in the initial placement of these loops and facilitate interpreting the effects of genomic variants.

After completion of the 3D molecular modeling work described above, an experimental structure for TASK1 (*KCNK3*) was released [34]. This protein is closer in sequence identity to TASK3 (62%) compared to the TWIK1 (*KCNK1*) and TRAAK (*KCNK4*) experimental structures used as templates in this study (27% and 28%, respectively) [26, 27]. In our model, all amino acids through Val243 are concordant with the TASK1 experimental structure. After Val243, our model was disordered and highly dynamic in simulations, concordant with the sequence not conforming to the C-terminal of TWIK1 or TRAAK. In TASK1-based models, Met249 faces into the membrane and we computed Met249Thr to be destabilizing, consistent in effect with our original analysis (see the “Results” section). Thus, our modeling was highly concordant with the TASK1-based model and we have interpreted variants using information from both.

An explicit molecular environment was generated using Visual Molecular Dynamics (VMD) [35] and CHARMM-GUI [36, 37]. An 80x80Å membrane bilayer patch of POPC was generated. The protein was oriented to the patch using sequence-based annotation of trans-membrane helices from UniProt and consensus annotations from MESSA [38]. Water was added to provide a 10 Å distance between TASK3 and environment boundaries. The environment was neutralized at 150 mM KCl by adding 75 K^+^ and 79 Cl^−^ ions. This initial explicit-environment system for WT TASK3 was used in further modeling, as described below.

### Structure-based calculations

We scored genomic variants by 3D structure-based algorithms. First, we generated dimer models of each genomic variant using FoldX [39, 40] version 4 for computational mutagenesis and calculating folding stability changes upon mutation (ΔΔG_fold_). The explicit-environment WT TASK3 system was modified for each genomic variant using the FoldX-generated models. We also assessed the initial models using Frustratometer [41], to quantify how each genomic variant changes local interactions, from a more integrated perspective that accounts for the many concurrent types of favorable and un-favorable interactions of the mutated residues.

### Molecular dynamics simulations

We studied the dynamics of *KCNK9* genetic variants on TASK3, and how they affect ionic interactions, using molecular dynamics (MD) simulation as implemented in the NAMD [42] software and the CHARMM27 force field [43]. Each protein model was analyzed in triplicate, beginning with energy minimization for 10,000 steps. Next, harmonic constraints were added to protein non-hydrogen atoms and the system was heated to 300 K over 300 ps using a Langevin thermostat. Protein constraints were gradually released over 1 ns. The entire system was simulated free of restraints for a further 32 ns. In total, we generated 110 ns of MD trajectory per variant. Across variants and replicates, we generated 2.3 μs of MD trajectory to study the effects of the variants in TASK3.

### Molecular dynamics analysis

We calculated root mean-squared deviation (RMSD) and root mean-squared fluctuation (RMSF) values using C^α^ atoms after structurally aligning each trajectory to the initial WT conformation and ignoring the mobile C-terminus. We used principal component analysis (PCA) to summarize the dominant conformational changes across trajectories. PCs were calculated in Cartesian coordinates using C^α^ atoms. We calculated the radial distribution function (RDF) for ions using VMD. The RDF of K^+^ describes the normalized probability of observing K^+^ ions within concentric shells. Electrostatic surfaces were sampled over time by extracting periodic frames from each simulation and assessed them each using APBS [33, 44]. Analyses were carried out using a custom structural bioinformatics workflow and leveraging the bio3d R package [45]. Protein structures and trajectories were visualized using PyMOL [45, 46] and VMD [35]. We generated our time-dependent prediction of effect for each genomic variant using a manual synthesis across multiple structure- and dynamics-based metrics as well as manual inspection of the simulation trajectories.

### Mammalian expression plasmids

Human TASK3 (Genbank^TM^ AF212829) cDNA, was cloned into the pcDNA3.1^+^ vector (Invitrogen, Carlsbad, CA, USA), gifted by Helen Meadows (GlaxoSmithKline, Harlow, UK) or into the pAcGFP1-N1 fluorescent vector (Clontech-Takara Bio Europe). Plasmids encoding for the human M3 muscarinic acetylcholine receptor (Genbank^TM^ AF498917) cDNA were obtained from the UMR cDNA Resource Center (Rollo, MO).

### Mutagenesis

Each of the clinically identified *KCNK9* variants (Arg131Ser, Arg131His, Arg131Pro, Met132Arg, Phe135del, Met156Val, Met159Ile, Phe164Cys, Thr199Ala, Tyr205Cys, Gly236Arg, Ala237Asp, Met249Thr, Ala320Thr) were introduced by site-directed mutagenesis into human TASK3 cDNA using the QuikChange kit (Agilent, CA, USA) as previously described [14]. Oligonucleotide primers were synthesized by Eurofins MWG Operon, Ebersberg, Germany, and all constructs sequenced by DNA Sequencing & Services, MRC/PPU, University of Dundee, Scotland.

### Cell culture

All experiments were performed using a modified human embryonic kidney 293 cell line, tsA201 (European Collection of Authenticated Cell Cultures; Sigma-Aldrich, UK), prepared and maintained as previously described [14]. Once cells reached 80% confluency, they were split and resuspended in media at a density of 7 × 10^4^ and 0.5 mL transferred to a 4-well plate containing a poly-D-lysine-coated coverslip, ready for transfection the following day.

### Transfection

Plasmids containing cDNA for either WT or a mutated TASK3 variant and a similar plasmid encoding the cDNA for green fluorescent protein (GFP) were co-transfected at a concentration of 0.5 μg using a modified calcium-phosphate protocol, as previously described [14]. The cells were then incubated at 37 °C in 95% O_2_ and 5% CO_2_ for 4–6 h, before being washed with phosphate-buffered saline. The cells were used for experiments 18–24 h later. All variants were expressed as homodimeric channels (each α-subunit of the dimer expresses the incorporated mutation) for all experiments. For experiments measuring the effect of G-protein coupled receptors, M3 receptors were also included in the transfection at a concentration of 0.5 μg.

### Whole-cell patch-clamp electrophysiology

Currents were recorded from GFP-fluorescing tsA201 cells expressing the cDNA of interest using whole-cell patch-clamp in a voltage clamp configuration and a step-ramp voltage protocol as previously described [14] using an extracellular solution composed of 145 mM NaCl, 2.5 mM KCl, 3 mM MgCl_2_, 1 mM CaCl_2_ and 10 mM HEPES (pH adjusted to 7.4) and an intracellular pipette solution of 150 mM KCl, 3 mM MgCl_2_, 5 mM EGTA, and 10 mM HEPES (pH adjusted to 7.4). All experiments were conducted at room temperature (20–25 °C), and currents were recorded using an Axopatch 1D patch clamp amplifier (Molecular Devices, Sunnyvale, CA), filtered at 2 kHz, digitized at 5 kHz. Extracellular control solution and modulatory compounds were perfused at a rate of 4–5 mL min^−1^.

### Electrophysiology data analysis and statistics

Data analysis of whole-cell outward current and analysis software was as previously described in Cunningham et al. [47]. Current-voltage graphs were obtained from the voltage ramp (− 120 mV to + 20 mV). Data were expressed as the mean ± 95% confidence intervals (CI), and *n* represents the number of individual cells recorded. Statistical analyses used were either a one-way ANOVA with a post-hoc Dunnett’s multiple comparisons test or an unpaired/paired Student’s *t*-test. Data was considered statistically different if *P* < 0.05 (*), *P* < 0.01 (**), *P* < 0.001 (***), and *P* < 0.0001 (****). Data from variants was compared with matched control data from WT TASK3 recorded either simultaneously or around the same calendar period and cell batch number.

### Confocal microscopy

Colocalization studies were performed according to the detailed methods outlined in Cunningham et al. [47]. In brief, WT human TASK3 and human TASK3_Tyr205Cys cDNA’s were subcloned into the pAcGFP1-N1 fluorescent vector (Clontech-Takara Bio Europe) to create a fusion construct with GFP and expressed in tsA201 cells using TurboFect transfection reagent (ThermoFisher, Loughborough, UK). Prior to fixing the cells, the plasma membranes of the cells were stained with CellMask Deep Red (CMR) (ThermoFisher, UK) and the nuclei with Hoechst 33528 (Sigma-Aldrich, UK). Coverslips of cells were mounted with Vectashield anti-fade mounting medium (Vector Laboratories, UK).

Confocal microscopy images were taken using a LSM 880 confocal microscope (Carl Zeiss, Oberkochen, Germany) and processed using Zen Black software (Carl Zeiss). Cells were excited with an argon laser at either 561, 488, or 405 nm for the CMR plasma membrane stain, pAcGFP fused channel and Hoechst 33528 stained nuclei, respectively.

Colocalization was determined using Zen Black software and Pearson’s correlation coefficient (PCC) was used to represent the degree of co-localization.

### Chemicals

Muscarine chloride, zinc chloride, dithiothreitol, 5-dithio-bis(2-nitrobenzoic acid), and methanethiosulfonates were all purchased from Sigma-Aldrich, UK. For high potassium (25 mM K) or acidic pH (pH 6.4) experimentation, the extracellular solution was adjusted accordingly. Muscarine chloride was added directly to extracellular solution at a concentration of 0.1 μM prior to use.

### Functional study correlation and variant pathogenicity classification

To appropriately weight the strength of functional evidence provided by computational modeling and in vitro electrophysiological studies, we generated a weighting scheme for each. For computational modeling, we primarily used four dynamic features as follows (see Additional file 2: Table S1): The “Δ3-HB bbn RMSD” is the change in protein backbone RMSD among the 3-helix bundles. Since the WT channel exhibits dynamics in simulations (median of 2.8 Å RMSD), we binned this metric into three levels based on how distorted the 3HBs were for each variant compared to WT: ≥ 0.3 ΔRMSD, within 0.3 ΔRMSD, and ≤ − 0.3 ΔRMSD compared to WT simulations. The “SSR of Filter C^α^ RMSF” is the sum of squared residuals for the selectivity filter alpha-carbon RMSF, between WT and each variant. We binned this metric into three levels compared to WT: greater than 1.0, between 1.0 and 0.5, and less than 0.5. The selectivity filter depends not only on the backbone, but also on sidechain orientations. So, we quantified the “Δside chain heavy atom RMSD TIG (Thr-Ile-Gly) motifs” binned into greater than 0.5, within 0.5, and less than 0.0. Finally, we used “anti-chamber open/close by PCs” to indicate if a single PC feature indicated opening the antechamber, two PC features indicated opening, no change, a single feature of more closed antechamber, and two features of more closed antechamber (Additional file 3: Video S1). For the electrophysiological studies, we used a two-step approach applying more weight to current than the other measured channel attributes. We first weighted each assay result: current amplitude (0–3 corresponding to the level of statistical significance (0: not significant; 1: 0.001 < *P* < 0.01; 2: 0.0001 < *P* < 0.001; 3: *P* < 0.0001) and regulation by GPCR or by pH (0: not significant; 1: *P* < 0.05) and added weights for each variant (max weight = 5). These were then rescaled to a 0–3 scale (0: sum weight = 0; 1: sum weight = 1; 2: sum weight = 2–3; 3: sum weight > 3). The only exception to this was the Tyr205Cys variant which had no measurable current but would have only received a sum weight of 3 since regulation by GPCR and pH could not be measured without any baseline current, but the damaging evidence was more significant than that weight alone.

Variants were classified according to the 2015 Guidelines by the American College of Medical Genetics and Genomics and Association for Molecular Pathology guidelines [48]. Per current ClinGen guidelines, PM2 was used as a supporting level of evidence [49]. Conservatively, PS2 was applied as a moderate level of evidence due to *KCNK9* being a paternally imprinted locus. PP3 was determined using CADD [50], MutationTaster [51], PrimateAI [52], SIFT [53], and Polyphen2 [54] (dbNSFP version 4.1 [55]) applying supporting level of evidence when 3 or more tools predict a damaging impact (Additional file 2: Table S1). The weight for functional evidence (PS3) was applied using only the electrophysiological data by the rescaled weight (none = 0; supporting = 1; moderate = 2; strong = 3). Agreement between computational and in vitro impact of variants was assessed using Spearman correlation and linear regression models.

## Results

### Diverse genomic variation in *KCNK9*

**Fig. 1:**
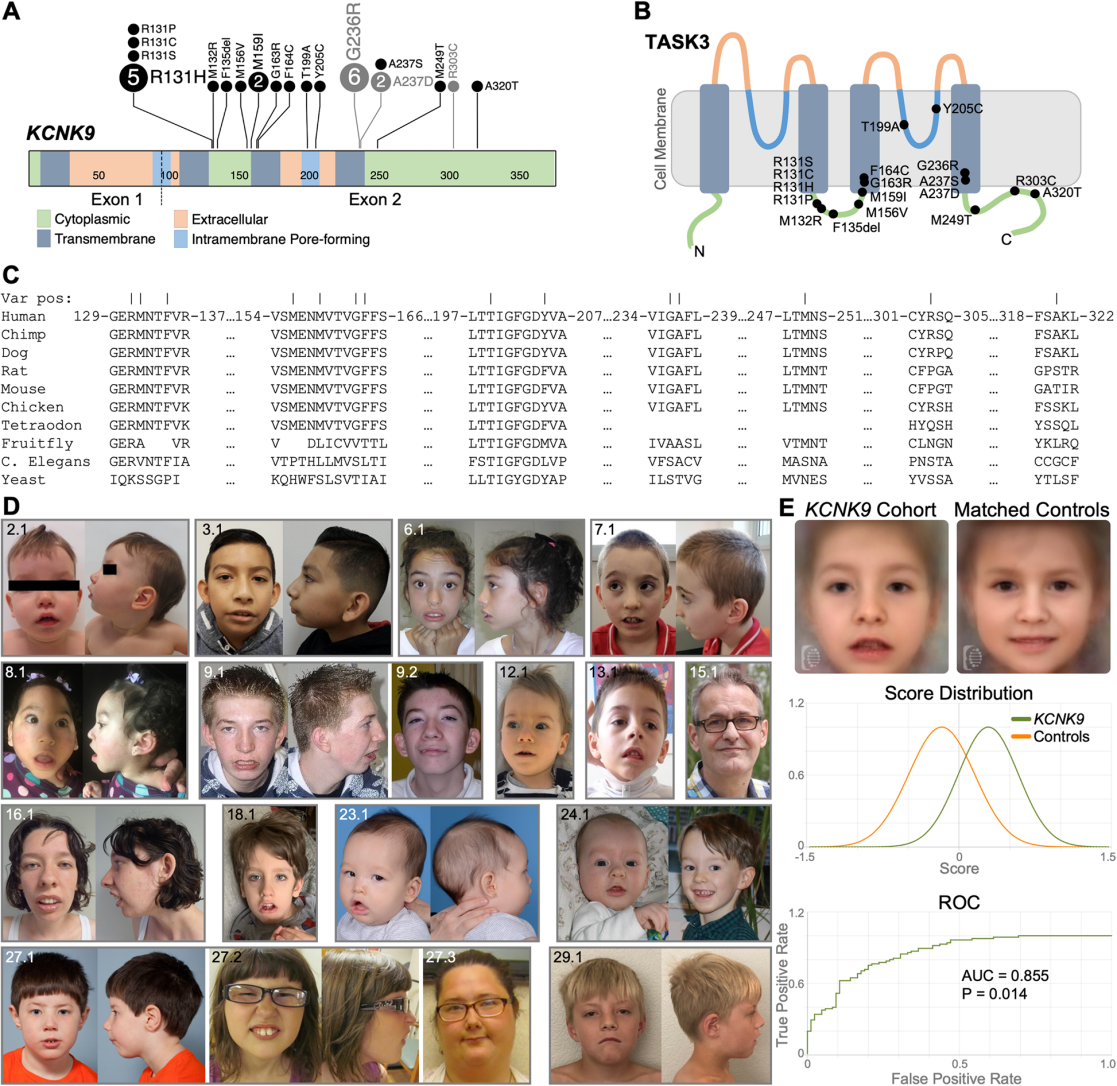
Individuals with *KCNK9* imprinting syndrome. **A** *KCNK9* coding exons showing all variants by predicted protein change using ProteinPaint (St. Jude Children’s Research Hospital). The number in the circle represents the number of families described with the variant, no number = 1 family. Previously published variants are gray. **B** TASK3 protein topology schematic showing the location of variants. **C** Conservation of residues affected by variants. Bars in top line indicate variant position. **D** Front and profile photos of individuals with *KCNK9* variants. The age at time of photograph is: P2.1: 1y 6 m, P3.1: 9y, P6.1: 10y 3 m, P7.1: 9y, P8.1: 1y 5 m, P9.1: 12y-18y, P9.2: 12y-18y, P12.1: 2y 2 m; P13.1: 6y 1 m, P15.1: 58y, P16.1: 17y, P18.1 (updated photo, Pt. 1, Graham et al. 2016): 6y, P23.1: 1y 3 m, P24.1: left 3 m, right 5y, P27.1: 6y 1 m, P27.2: 8y1m, P27.3: 28y, P29.1: 8y 1 m. y = years; m = months. **E** Facial analysis using Face2Gene Research application (FDNA Inc. Boston, MA) of individuals with *KCNK9* imprinting syndrome (frontal photos of P3.1, P6.1, P7.1, P8.1, P9.1, P12.1, P13.1, P16.1, P18.1, P19.1*, P20.1*, P21.1*, 23.1, P24.1 (3 m photo), 25.1*, 27.1, and 29.1 compared to age, sex, and ethnicity matched controls. The aggregated binary comparison (AUC = area under the curve, ROC = receiver operating characteristic) demonstrates a significant difference between the two cohorts (*P* < 0.05). *used published photos

This study describes 47 affected individuals with heterozygous *KCNK9* variants from 29 families. We summarize the originally described Arab-Israeli kindred of 15 affected individuals and six previously published singletons in addition to the 26 newly identified individuals from 22 families harboring 15 unique, novel *KCNK9* variants (Fig. 1A).

*KCNK9* is paternally imprinted, allowing pathogenic variants to be present on the paternal allele without causing disease, and thus could be found in healthy populations. We observed that *KCNK9* is nearly intolerant to missense variation with a gnomAD (v2.1.1) [56] missense constraint *Z*-score = 2.9 (observed/expected ratio = 0.48; CI 0.41–0.56) where significant constraint is *Z* > 3.09. *KCNK9* is less constrained than other genes causing dominant neurodevelopmental disorders [57], however, and 9/19 variants (Additional file 2: Table S1) fall in constrained coding regions (CCR) > 95th percentile [58]. The identified *KCNK9* variants (Fig. 1A, B and Additional file 2: Table S1) are absent or extremely rare in the population and fall either within the transmembrane (TM) or the intracytoplasmic domains, without occurring in the extracellular regions of the protein, and most affect highly conserved residues (Fig. 1C). Six families carry the p.(Gly236Arg) variant affecting 20 individuals, a known alteration hotspot. Among the novel variation identified in our cohort, eight families with nine affected individuals carry missense alterations at Arg131, including five families with p.(Arg131His), and one each with p.(Arg131Cys), p.(Arg131Pro), and p.(Arg131Ser) in an affected sibling pair. Two siblings from an additional family have the neighboring residue modified by a p.(Met132Arg) variant. Additionally, two unrelated probands have a p.(Met159Ile) variant. The remaining variants identified were unique to each family. Congruent with *KCNK9* paternal imprinting, ten families demonstrated maternal inheritance and in 16 families the variant occurred de novo in the affected individual or the mother (Additional file 2: Table S1). Thus, we have identified diverse genetic alteration of TASK3 channels associated with KIS that lacked mechanistic understanding and disease association, motivating the functional characterization of them.

### Spectrum and variability of clinical phenotypes associated with *KCNK9* variation

By assessing the available clinical data, we describe the phenotypic spectrum and variability across our cohort (Table 1). Detailed clinical histories are provided in the Additional file 1: Supplementary Note. Most individuals presented with neurodevelopmental delays, ID, hypotonia, behavioral abnormalities, and facial dysmorphism.

**Table 1:**
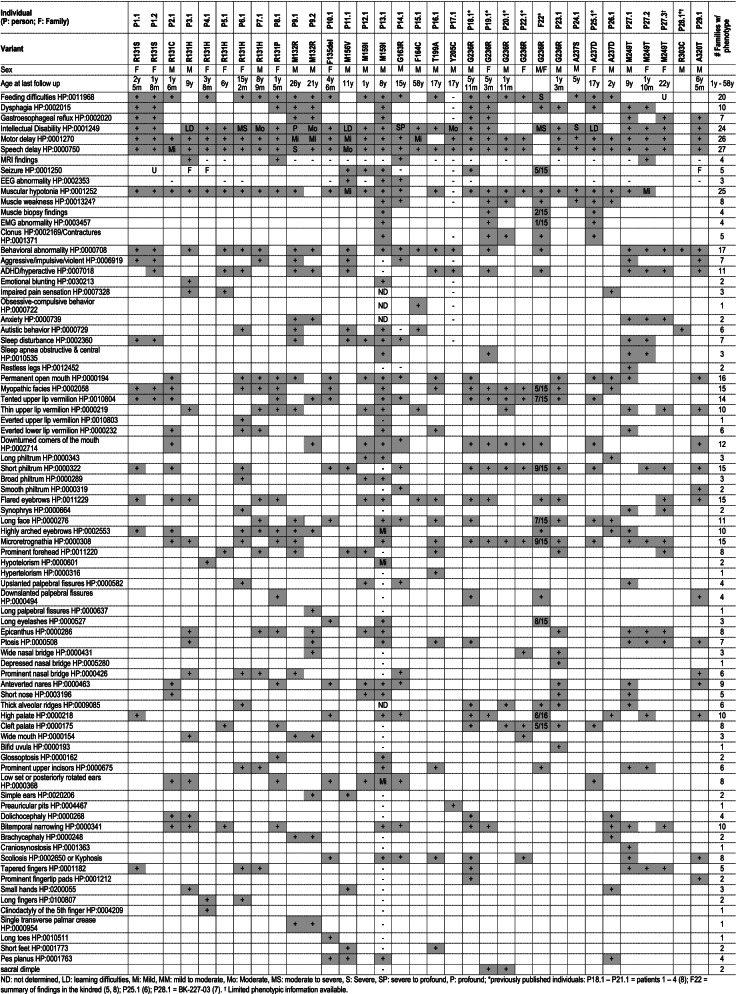
Phenotypes of individuals with KCNK9 variation

Neurologically, the majority of individuals were affected with mild to severe profound ID (*n* = 24 families, 83%) and motor (*n* = 26, 90%) and speech (*n* = 27, 93%) delays with mild to profound muscular hypotonia (*n* = 25, 86%) especially during infancy. This was often accompanied by myopathic changes on muscle biopsy and electromyography (EMG). Behavioral and cognitive abnormalities were common (*n* = 17, 59%) and included attention deficit hyperactivity disorder (ADHD), autism spectrum disorder (ASD), and aggressive, impulsive, or violent behavior. For example, proband P6.1 displayed autistic features with stereotypies (Additional file 4: Video S2).

Afebrile seizures were reported in five families (17% of families, Additional file 5: Table S2). Five of 15 affected individuals from family F22 reportedly developed seizures, but without further detail [5, 8]. The four additional probands (P11.1, P12.1, P13.1, and P18.1) who reported afebrile seizures had seizure onset between 2 and 8 years of age. Seizures were well controlled with medication (single or combination therapy including lamotrigine, sodium valproate, clonazepam, or oxcarbazepine). In three of these individuals, febrile seizures and unconfirmed episodes concerning for seizures preceded afebrile seizures with onset as early as 1 month of age. Three additional individuals (P3.1, P4.1, and P29.1) reported febrile seizures only and P1.2 experienced episodes of suspected absence seizures that were not confirmed.

Dysmorphic features were reported in all individuals with data available (Table 1 and Fig. 1D). Analysis of facial photos (Fig. 1E) of 17 unrelated individuals with KIS compared to matched controls using unbiased facial recognition software [21] demonstrated a statistically significant difference in dysmorphic facial features across our cohort (*P* = 0.014, AUC = 0.88 ± 0.05). The composite facial gestalt image demonstrates elongated face, arched eyebrows, downturned corners of the mouth, thin upper lip, tented upper lip, short philtrum, and bitemporal narrowing. Additional features including thick alveolar ridges, cleft or high palate, and tapered fingers were relatively common. Thus, these descriptions extend and refine the spectrum of signs and symptoms that characterize this syndrome.

### TASK3 variants display altered molecular mechanics and dynamics properties

**Fig. 2:**
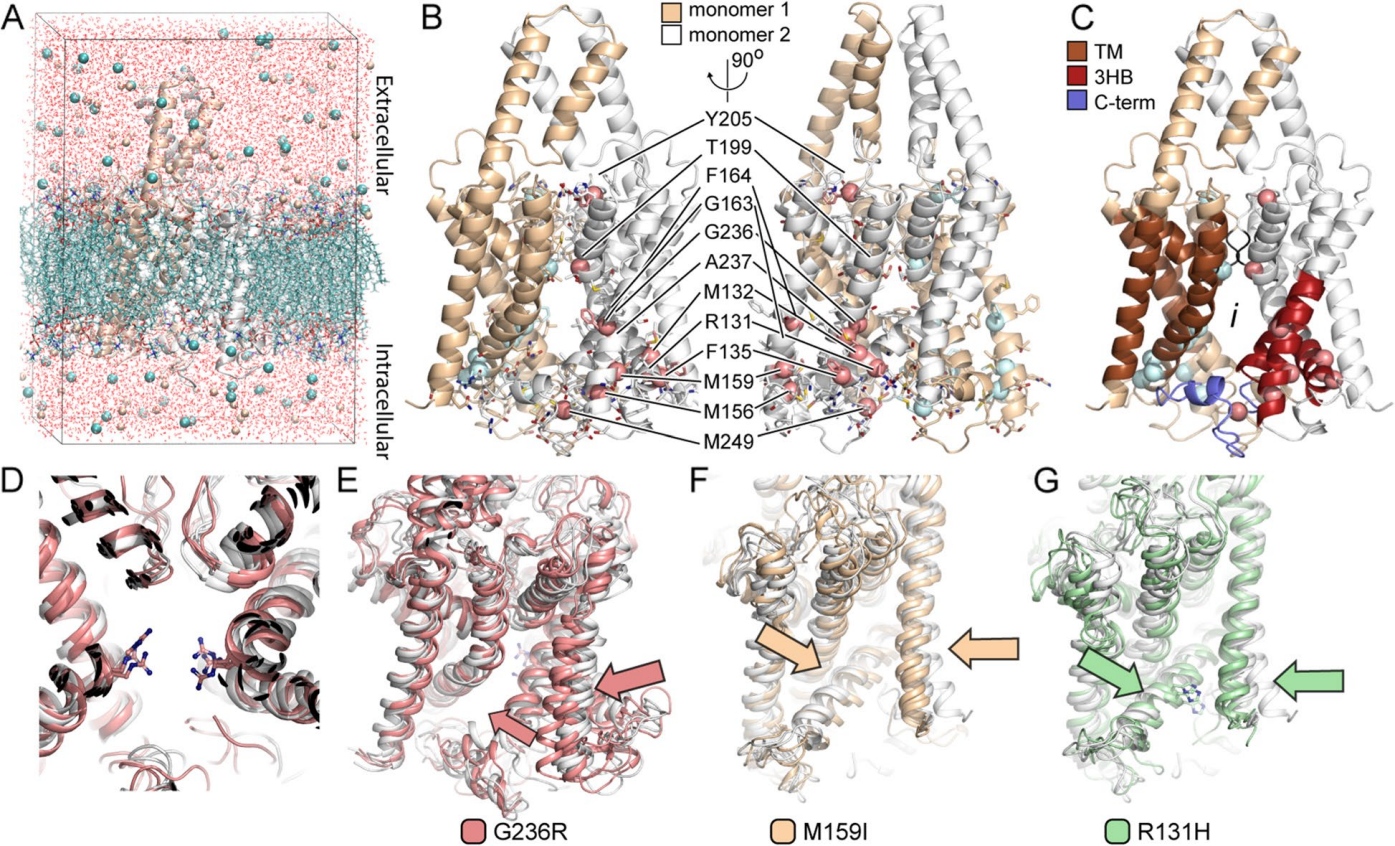
TASK3 structure and variant locations. **A** We modeled TASK3 in an explicit environment and show the dimer colored by monomer. We omit the environment from other images for simplicity. **B** We mark the locations of variants observed in our cohort. Pink spheres mark sites of variants in one monomer and cyan sphere in the other. R303C and A320T fall outside of the modelable region. **C** We show the same view of TASK3, colored by features of the structure that we will use in describing the effects of genomic variants. We colored only one monomer since features overlap. The trans-membrane (TM) helices form the core of the structure and three of them form a type of 3-helix bundle (3-HB). TIG motifs form the lower section of the selectivity filter and are colored black. The C-terminus is predicted to fold along the intracellular-facing side and may participate in a gating mechanism. Most variants are on the intracellular-facing side or facing into the interior central chamber, indicated by an *i*. **D** G236R places positively charged Arginine side chains facing inside of the channel, not only occluding it but likely adding a cation blockade. **E** From our MD simulations, we also observed consistent conformational changes associated with G236R. **F** The variant M159I, which occurs within the 3-HBs, was also associated with a consistent change in N-terminal helix orientation. **G** Three different variants of R131 are observed and have effects on 3-HB organization and orientation relative to the N-terminal helix

**Fig. 3:**
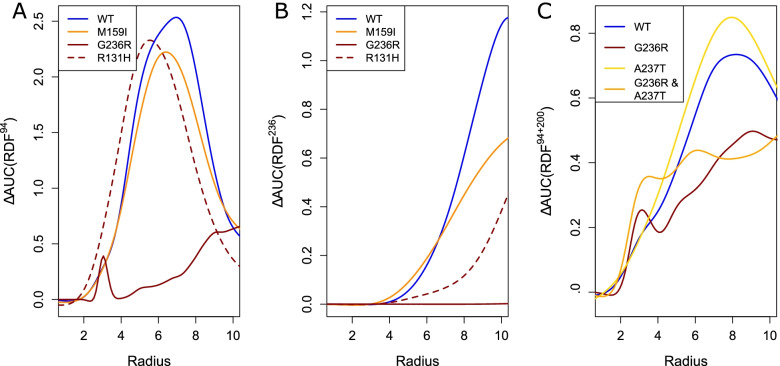
Functional alterations in TASK3 are associated with altered ion distribution. We show, here, selected examples with broader characterization in Additional File 6: Fig. S6 and S7. **A** We show smoothed K^+^ RDF for selected variants, with M159I and R131H showing WT-like but lower presence of K^+^ around the selectivity filter and a depletion for G236R. **B** The smoothed K^+^ RDF centered at residue 236, which is towards the cytoplasmic face, again shows a drastic depletion for G236R, less drastic for R131H, and a more modest change for M159I. **C** Smoothed K^+^ RDF centered around the selectivity filter shows that the significant depletion for G236R is partially rescued by the A237T double-variant with a modest increase for A237T alone. This is consistent with previously reported experimental assays that showed A237T to be a partial compensating variant for G236R

We used models of 3D TASK3 channels (Fig. 2A–C), leveraging experimental data from the gene family, in calculations of a variant’s effect on the protein (*N* = 14; Table 2). Protein structural modeling in 3D enables calculations of specific biochemical and biophysical details. Such calculations provide information that is distinct from genomics algorithms [59] and enable us to evaluate variant impact on channel mechanics. Using the 3D models, we simulated TASK3 dynamics (Additional file 6: Fig. S1), which were consistent across technical replicates (not shown) and revealed mutation-specific mechanics (Fig. 2D-G; details in Additional file 1: Supplementary Note). Gly236Arg indeed occluded, placed positive charge within the channel antechamber (Fig. 2D) and distorted the selectivity filter (Additional file 6: Fig. S2A). The selectivity filter is characterized by a ring of hydrophobic and aromatic residues that form interactions between monomers and facilitate transport through the channel (Additional file 6: Fig. S2B). Simulations also showed rearrangements of the selectivity filter for Arg131His, while Met132Arg led to blockage of mutated channels and Phe135del distorted both regions, supporting an interpretation of reduced function (Additional file 2: Table S1). The remaining mutated channels, other than Phe164Cys and Ala237Asp, showed a more open channel interior in simulations (Table 2), which we interpreted as being more permissive to ion transport. Some variants simultaneously showed features that could associate with being less permissive, such as Arg131His which had a distorted selectivity filter, and Arg131Ser which had a narrower channel entrance. To better summarize aggregate effects across these protein dynamics changes, we quantified changes to K+ distribution at key locations (Additional file 6: Fig. S3 and S4) and found severe depletion of K^+^ nearby the selectivity filter for Gly236Arg (Fig. 3A). At the cytoplasmic face, Gly236Arg showed nearly no K^+^, Arg131His showed little, and Met159Ile comparatively more but still diminished compared to WT (Fig. 3B). Simulations even demonstrated the partial rescue of Gly236Arg by Ala237Thr co-mutation, previously shown experimentally [14] (Fig. 3C and Additional file 1: Supplementary Note), supporting the mechanistic value of the data. Interestingly, [K^+^] around the selectivity filter and ΔΔG_fold_ were significantly correlated (*ρ* = 0.50), indicating a link between our structure- and dynamics-based calculations. Together, blinded to the electrophysiology data (below), simulations predicted loss of function for Gly236Arg, Phe135del, and Met132Arg, little change for Phe164Cys and Ala237Asp, likely gain of function, but with a mix of gain- and loss-associated features, for Met156Val, Arg131His, Arg131Ser, and gain of function for Arg131Pro, Met159Ile, Thr199Ala, and Met249Thr (Table 2). Thus, 3D structural and time-dynamic simulations enriched the information available from genomics, for explaining mutated TASK3 channel mechanics.

**Table 2:** *KCNK9* variants in this study assessed for TASK3 structure- and dynamics-based changes

Protein variant	ΔΔG_**fold**_	ΔFour-body Pot.	ΔSRLF	ΔPC ^**a**^	Transport events ^**b**^	ΔElec surface	Selectivity Filter [K^**+**^] ^c^	Δ[K^**+**^] ^c^	Blinded prediction ^d^
R131S	1.36 ± 0.17	− 2.63	1.13	PC2 (2.6σ)	8	+	0.39	–––	Occluded-permissive
R131H	1.05 ± 0.17	− 1.48	1.26	n.s.	4	+	1.42	–	Mixed-permissive
R131P	6.87 ± 0.65	4.34	− 0.71	PC2 (− 1.1σ) PC3 (1.5σ)	10	--	1.08	––	Mono-permissive
M132R	2.74 ± 0.10	14.77	− 1.21	PC3 (− 1.1σ)	14	n.s	2.30	+	Mixed-permissive
F135del	NA	NA	NA	PC3 (− 2.4σ)	9	n.s	1.52	–	Mixed-permissive
M156V	2.54 ± 0.17	− 3.75	1.22	n.s.	6	+	0.62	–––	Permissive
M159I	8.01 ± 0.52	− 22.18	0.86	n.s.	8	+	1.93	n.s	Permissive
F164C	3.48 ± 0.43	− 31.18	− 0.55	PC1 (1.2σ)	8	n.s	2.92	++	Permissive
T199A	0.92 ± 0.14	1.83	0.08	PC2 (− 2.7σ) PC3 (2.3 )	12	+	1.93	n.s	Permissive
Y205C	4.01 ± 0.63	− 5.57	2.94	n.d.	n.d.	n.d.	n.d.	n.d.	n.d.
G236R	− 2.59 ± 0.54	− 3.41	− 0.46	PC2 (− 2.8σ) PC3 (1.6σ)	0	++	0.25	–––	Occluded
G236R & A237T	− 2.40 ± 0.54	0.32	NA	PC1 (− 2.0σ) PC3 (− 2.4σ)	10	++	0.81	––	Occluded-attenuated
A237D	5.36 ± 0.17	13.51	− 1.85	PC2 (− 1.3σ)	8	n.s	1.37	–	Permissive
A237T	0.13 ± 0.07	− 0.60	− 0.76	PC2 (− 2.9σ)	4	+	1.80	n.s	Moderately permissive
M249T	6.41 ± 0.83	5.09	0.59	PC2 (− 3.1σ) PC3 (1.2σ)	1	--	2.00	n.s	Permissive

*SRLF* single residue level frustration, *NA* not applicable, *n.d.* not determined

^a^ PC alterations of at least 1 standard deviation (σ) away from WT are noted; others are labeled as not significant (n.s.); data used ignored the C-terminus (see Methods)

^b^ Compared to a WT value of 2 transport events

^c^ Defined based on the K^+^ radial distribution functions (Fig. S4 and S5) at 6.9 Å from residues 94 and 200. Comparisons are to the WT value of 1.8

^d^ Using only the computational simulation data, we predict if the variants will impair potassium transport or not

### Electrophysiological studies reveal altered current by TASK3 variants

**Fig. 4:**
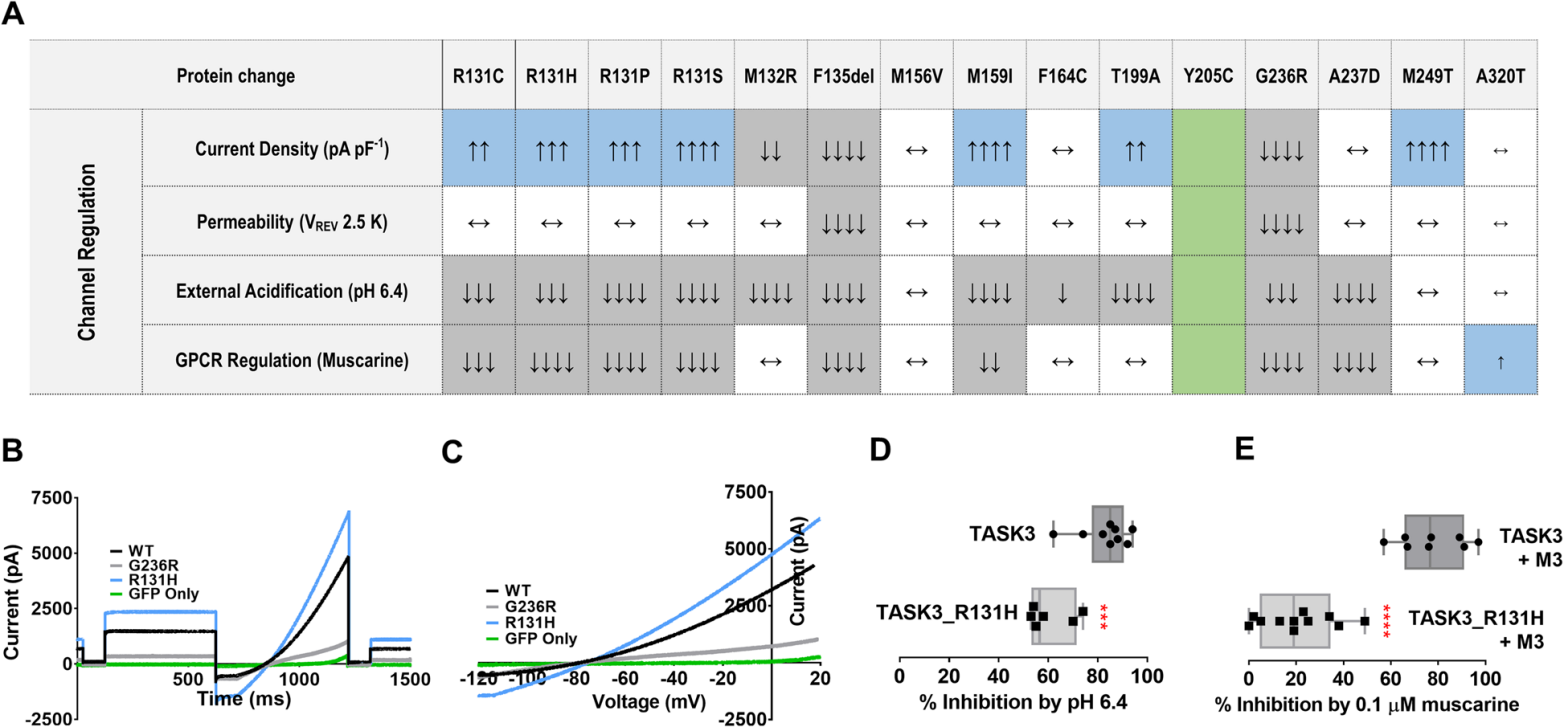
Effect of individual protein changes on channel regulation by various modulators. **A** Boxes highlighted blue denote an increase in the measured parameter compared with matched WT controls where ↑*P* < 0.05, ↑↑*P* < 0.01, and ↑↑↑*P* < 0.001, and ↑↑↑↑*P* < 0.0001, determined using an unpaired Student’s *t*-test. Boxes highlighted gray denote a decrease in the measured parameter compared with WT, where ↓ *P* < 0.05, ↓↓*P* < 0.01, ↓↓↓*P* < 0.001, and ↓↓↓↓*P* < 0.0001. White boxes signify no change in effect from WT (←→*P* ≥ 0.05). Green boxes signify non-functional variants and mirror GFP-only transfected cells. **B** Current amplitude vs time. **C** Current amplitude vs Voltage. **D** Inhibition by acidic pH. **E** Inhibition by muscarine. ***P < 0.001 and ****P < 0.0001 for between group differences, determined using an unpaired Student’s *t*-test. Square or circle symbols represent individual data points for each condition and channel. Error bars represent 95% confidence intervals. pA, picoamp; pF, picofarad; V_REV_, reversal potential; ms, millisecond; mV, millivolt; M3, muscarinic receptor 3

To evaluate the functional impact of *KCNK9* variants on TASK3 channel properties, we performed whole-cell patch clamp electrophysiology to record currents passing through 15 clinically identified *KCNK9* channel variants, transiently expressed in human tsA201 cells and characterize their properties, (Additional file 7: Table S3). The KIS variant, Gly236Arg, introduces a positively charged arginine in the TM4 region of the channel and gives significantly reduced (*P* < 0.05) and inwardly rectifying currents [14]. For 13 novel variants (Arg131Ser, Arg131Cys, Arg131His, Arg131Pro, Met132Arg, Phe135del, Met156Val, Met159Ile, p.Phe164Cys, Thr199Ala, p.Ala237Asp, Met249Thr, p.Ala320Thr), however, the opposite effect—outwardly rectifying pico-ampere (pA) currents—was observed, determined by ramp changes in holding potential from − 120 to + 20 mV (Fig. 4A–C and Additional file 7: Table S3). Seven of these variants (Arg131Ser, Arg131Cys, Arg131His, Arg131Pro, Met159Ile, Thr199Ala, Met249Thr) had significantly increased outward currents (*P* < 0.05, unpaired *t*-test), while Met132Arg and Phe135del, located on the intracellular cytoplasmic loop between TM2 and TM3, had significantly (*P* < 0.05) reduced outward currents (Fig. 4A and Additional file 7: Table S3), compared to experimentally matched WT controls. Interestingly, the remaining four variants (Met156Val, Phe164Cys, Ala237Asp, Ala320Thr) were functionally indistinguishable from WT in this assay (Fig. 4A). Twelve variants had mean zero current potentials similar to WT (*P* > 0.05) and close to the equilibrium potential for K^+^ under these recording conditions (Fig. 4A–C). The Phe135del variant was the only variant that displayed a significant (*P* < 0.05) shift in reversal potential to become more depolarized, similar to that observed with Gly236Arg. Tyr205Cys occurs in the second pore (P2) region of the channel and was non-functional under our experimental conditions, with no measurable current and zero current potentials (Additional file 7: Table S3), indistinguishable from green fluorescent protein (GFP)-only transfected cells. Using fluorescently tagged channels in confocal microscopy of WT, Gly236Arg, and Tyr205Cys channels, we found that Tyr205Cys channels had similar TASK3 protein levels at the plasma membrane as WT channels (Additional file 6: Fig. S5), suggesting the Tyr205Cys variant is trafficked as efficiently to the plasma membrane as WT. Attempts to restore current through Tyr205Cys channels using cysteine-modifying agents (dithiothreitol (DTT), 5″-dithio-bis(2-nitrobenzoic acid) (DTNB or Ellman’s), or methanethiosulfonates (MTS)) were unsuccessful (Additional file 6: Fig. S6). Thus, the novel variation studied was dissimilar to Gly236Arg and significantly different in current density from WT, supporting its role in defining KIS but with a distinct underlying mechanism.

### Alterations in TASK3 regulation is a dysfunctional feature shared by KIS variants

Sensitivity to extracellular pH changes is a hallmark regulatory fingerprint for TASK3 channels. With a pKa of 6.7, TASK3 channels are inhibited by increased extracellular acidification from physiological pH [1, 60, 61]. Interestingly, Gly236Arg mutated channels demonstrate reduced sensitivity to extracellular acidification (Additional file 8: Table S4 and see Veale et al. [14]). By determining the pH sensitivity of the novel TASK3 variants, we find that ten of the 13 variants had decreased sensitivity to extracellular acidification from pH 7.4 to 6.4 (Fig. 4A, D and Additional file 8: Table S4) similar to Gly236Arg channels; we found no change in pH sensitively with Met156Val, Met249Thr, or Ala320Thr. Tyr205Cys could not be measured due to a lack of measurable current at − 40 mV. These data show that most variants are associated with a similar disruption of channel regulation by extracellular acidification as the Gly236Arg channels.

Notably, TASK3 channels are also regulated by activated GPCRs leading to inhibition, and Gly236Arg channels show complete loss of G_αq_-mediated channel inhibition (Additional file 9: Table S5, and see Veale et al., 2007, 2014) [13, 14]. Thus, we assayed the loss of G_αq_-mediated channel inhibition in the presence of 0.1 μM muscarine and found that seven of the 13 channels (Arg131Ser, Arg131Cys, Arg131His, Arg131Pro, Phe135del, Met159Ile, Ala237Asp) exhibited significantly (*P* < 0.05) reduced G_αq_-mediated inhibition (Fig. 4A, E and Additional file 9: Table S5) similar to Gly236Arg, whereas Ala320Thr exhibited increased sensitivity to G_αq_-mediated inhibition. The five remaining variants, (Met132Arg, Met156Val, Phe164Cys, Thr199Ala, Met249Thr), retained a sensitivity to G_αq_-mediated inhibition similar to WT controls (Fig. 4A). These studies show that activated GPCR regulation of TASK3 channels is altered by a majority of clinical variants, similar to Gly236Arg, and suggest channel regulation as a defining functional consequence of clinical *KCNK9* variation associated with KIS.

### Integrated computational and experimental assessment of altered channel mechanisms

We assessed the congruency of the computational and experimental results and show the computational assessment, blinded to in vitro data, was concordant with in vitro data. We categorized the in vitro and computations evidence, each, into the following weights of impact (Additional file 2: Table S1; see methods for details): none (no change from WT; weight = 0), supporting (differences are observed, but clinical significance is unclear; 1), moderate (function is likely impaired; 2), and strong (function is altered consistent with being disease causal; 3). All variants computationally predicted to occlude the channel were of strong impact, experimentally. Three of the four variants with computationally modeled features showing the channel being both more- and less-permissive were of strong impact (the fourth was of moderate impact), experimentally. Of the remaining six variants, four were of moderate impact and one each of supportive and strong impact, experimentally; among these six, computations were concordant with in vitro impact class but predicted a greater impact for Met156Val and Ala237Asp and a lesser impact for Met159Ile and Ala320Thr. Met156Val is unique among the in vitro tests for no measured impact on intrinsic function or regulation. A further three, Phe164Cys, Ala237Asp, and Ala320Thr, have minimal-to-no change in intrinsic protein function but do alter how it is regulated. Importantly, our computational models were not setup to test changes in channel regulation. Finally, Met159Ile has among the most damaging structure-based scores (Table 2). Thus, while we see fewer deformations of the Met159Ile channel in simulations, Met159Ile is predicted to alter the structure of TASK3. Therefore, the biochemical and mechanical link between our simulations and in vitro data indicates their mutual value for interpreting the effects of genetic variants.

Then, we further datamined our simulations for which features associated best with experimental results. We found that alterations of position for the TIG motif that defines the selectivity filter and 3-Helix Bundle (3-HB) organization appear to be the most consistent structural features that associate with current amplitude changes (Additional file 2: Table S1). Four variants (Met132Arg, Phe135del, Gly236Arg, and Tyr205Cys) demonstrated decreased or no current amplitude experimentally. By computations, these four all showed greater distortion of 3-HB, compared to WT. Concurrently, Met132Arg and Phe135del had a more closed antechamber and Gly236Arg a more open antechamber. Thus, these four variants are predicted to be more conformationally unstable, leading to loss of amplitude. Six variants (Arg131Ser, Arg131His, Arg131Pro, Met159Ile, Thr199Ala, and Met249Thr) have increased amplitude by electrophysiology. All six except Arg131Ser had less distortion of 3-HB. Concurrently, Arg131Pro and Met249Thr have a more open antechamber, and Arg131His/Ser had a more closed antechamber. TIG motifs are significantly altered for all six except for Arg131Pro. Thus, these variants were overall more conformationally stable, but TIG alteration indicated a loss of conformational control over ion movement. Three variants (Met156Val, Phe164Cys and Ala237Asp) demonstrated normal WT current amplitudes in electrophysiological assays. These three showed varied alteration of the 3-HBs and TIGs in simulations. A large 3-HB alteration was calculated for Ala237Asp, although no affect was seen on current amplitude, unlike the gain-of-function Ala237Thr synthetic variant [14, 62]. Deviation of the TIGs was minor for Phe164Cys and Met156Val but large for Ala237Asp. These three variants have mixed 3D features that support an overall neutral to modest increase in amplitude, concordant with our experiments. Thus, structural simulations and functional experiments provided inferences on how mutated TASK3 channels may become dysfunctional.

### Correlation of functional properties of KIS variants and pathogenicity classifications

**Fig. 5:**
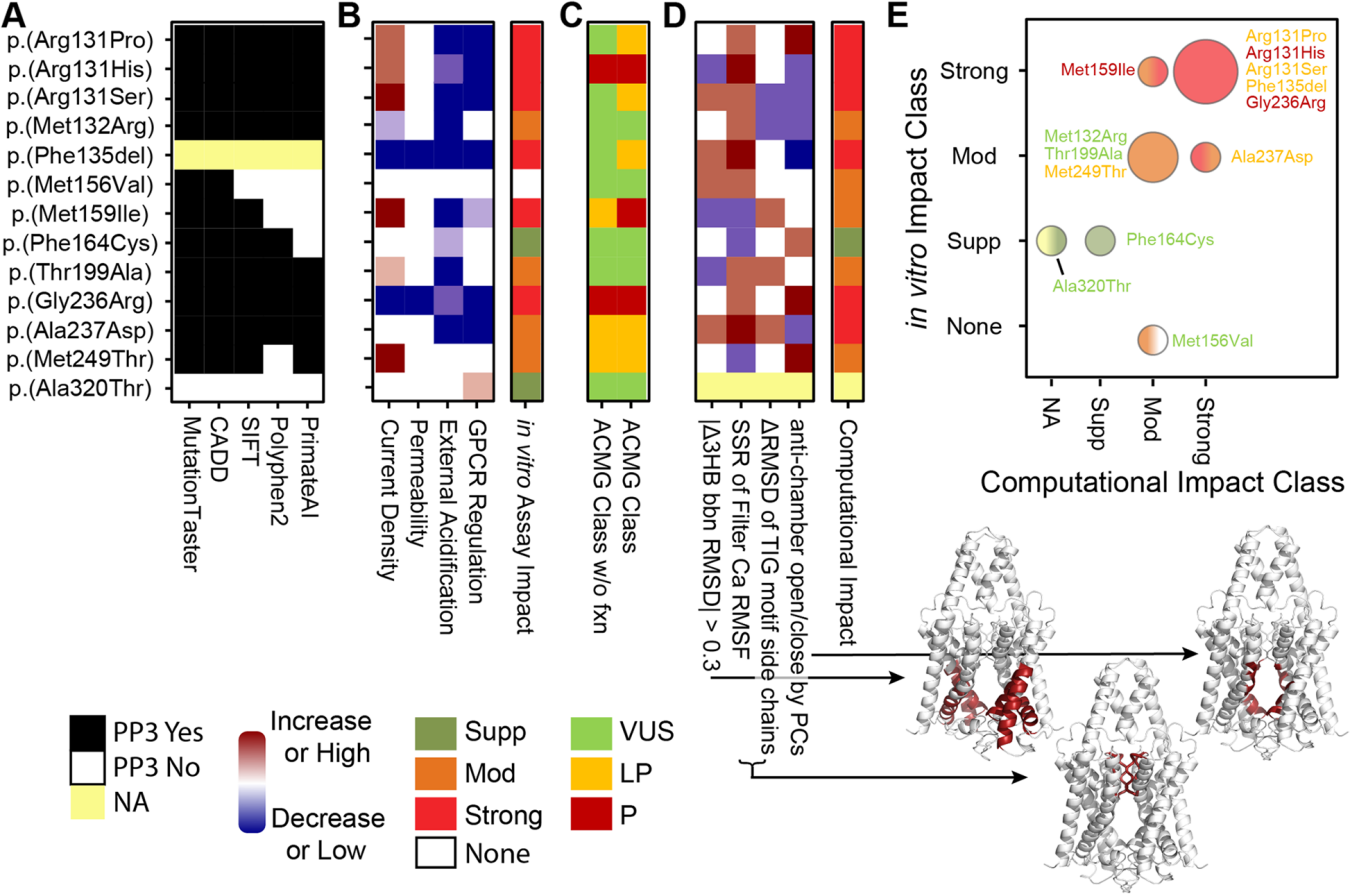
Combining experimental and computational approaches adds mechanistic detail to interpreting genetic variants. **A** Most DNA sequence-based algorithm predicts our cohort’s variants uniformly, but there is a stark lack of consensus among them. Each genomic score was thresholded according to how we used them to the ACMG classification PP3 criteria. **B** Our experiments clarified specific functional changes for each mutated protein, resulting in an in vivo impact class and **C** updating the ACMG classification of each variant. **D** Computational assays were additionally summarized and demonstrated variant-specific changes to key regions of the protein and resulting in a predicted impact class (further detail in Additional file 2: Table S1). **E** The impact classes from experimental and computational approaches were highly concordant, demonstrating the potential for computational tools to enhance the information available for interpreting genetic variants. We summarized concordance using a bubble plot with radius proportional to the number of variants in each class. Variants are colored according to their ACMG Class, and bubbles are colored according to Impact Class (left side computational and right in vitro)

We next sought to classify the *KCNK9* variants using a 5-point pathogenicity scale from benign to pathogenic using all relevant and available data for each variant. DNA-based predictive algorithms were nearly unanimous in the variants being damaging, save p.(Met156Val) and p.(Ala320Thr) (Fig. 5A). Our in vitro data was more nuanced and demonstrates that each variant can have unique features, but that channel regulation was most consistently disrupted (Fig. 5B). To gauge the strength of evidence supporting a damaging interpretation for the in vitro heterologous cell-based studies, and for the computational modeling studies, we used the four-point weights of impact calculated above (Additional file 2: Table S1; see methods for details). To classify the *KCNK9* variants in terms of pathogenicity, we used the current American College of Medical Genetics and Genomics and Association for Molecular Pathology guidelines [48] (Fig. 5C and Additional file 2: Table S1). Therefore, concordant with these guidelines, we considered only the in vitro cell-based studies as contributory for PS3 and applied the most weight to current amplitude (see methods for details). Using existing data, four of the 19 *KCNK9* variants would be classified as pathogenic, seven as likely pathogenic, and eight remained VUS. The in vitro data we gathered in this study promoted three VUS to likely pathogenic and one likely pathogenic to pathogenic (Fig. 5C). Computations also demonstrated nuanced patterns, which we summarized above using distortion of the 3-HBs and TIGs (Fig. 5D). The variants had a very similar strength of evidence supporting a damaging impact on the protein from both experimental and computational approaches (Fig. 5E; rank correlation ρ = 0.72, *P* = 0.0041). Thus, the interpretation of our data in terms of these guidelines provides information of immediate application to clinical medicine, yet nuances from computational and in vitro experiments indicate that information with more detailed mechanistic value can improve the interpretation if KIS genetic variants.

## Discussion

The current study makes significant contribution to the understanding of the field of channelopathies in general and to KIS, in particular, by characterizing a sizable cohort of individuals with novel genetic variants in *KCNK9* affected with this neurodevelopmental syndrome. In addition, we comprehensively characterize the functional impact of the variants using a multi-tiered approach. We report 26 previously unpublished individuals with 15 novel variants displaying phenotypic traits similar to those with the previously reported Gly236Arg variant associated with KIS. Moreover, we report the existence of two mutational hotspots in *KCNK9*, occurring at the previously reported Gly236 and the newly identified Arg131 codons. Computational protein modeling, molecular mechanic calculation, molecular dynamics simulations, and whole-cell patch-clamp electrophysiological techniques, when combined, strongly suggest that the large majority of these variants alter channel function and herein we classified them as pathogenic or likely pathogenic for KIS.

Fortunately, assembling this large mutational landscape for KIS allowed us to perform detailed clinical phenotyping of 47 affected individuals from 29 families with this syndrome (Table 1). The predominant features of this disorder include motor and speech delay, ID, early feeding difficulties, muscular hypotonia, behavioral abnormalities including hyperactivity and aggression, and dysmorphic features including an elongated face, permanent open mouth, microretrognathia, high arched eyebrows, and bitemporal narrowing. Less common features include seizures that can develop in childhood and were mostly controlled by medications, combined obstructive and central sleep apnea, and scoliosis. These features are consistent with the originally described p.(Gly236Arg) variant, but with a wider range of severity. Other syndromes that might be considered in the differential diagnosis include 22q deletion syndrome, Cohen syndrome, and Prader-Willi syndrome. The constellation of clinical features observed in KIS is not specific enough to make this disorder easily recognizable in the clinic, and it will therefore require a molecular diagnosis to confirm.

Unlike the original Gly236Arg mutant channels, which have reduced inwardly rectifying current [14], over 50% of the newly described variants had significantly increased outward currents. Notably, although the novel KIS variants had variable effects on channel current, they had a much more consistent impact on TASK3 regulation by extracellular pH and also by activated GPCRs. Taking into consideration general principles of protein dynamics, genetic variants can shift the balance of motions and thereby alter protein function [63, 64]. Variants that occur at sites within the lower 3-HB (Arg131, Met132, Phe135, Met156, Met159, and Ala237) likely alter hydrophobic packing of the region giving an increased probability of K^+^ near the channel pore and more K^+^ transport events. This is in stark contrast to the decrease seen with Gly236Arg, suggesting distinct channel dysfunction by these novel variants. In particular, genetic variants at position 131 lead to different extents of distortion that pull the N-terminal helix inward while also pulling the 3-HB helices outward. These changes displace the C-terminus of the other monomer from its normal interaction, and it shifts inwards into the ion transport cavity. This shift is greatest for Arg131His with Arg131Pro and Arg131Ser having more variability between them. Thus, we believe distortion of the 3-HB configuration could provide a novel metric for tracking the disruption of the channel by genomic variants.

The Tyr205Cys variant occurring immediately after the conserved ion selectivity filter sequence (GFG) in the second pore region of the channel resulted in completely non-functional channels. This loss of function is most likely the consequence of replacing an amphipathic tyrosine with a hydrophobic cysteine residue in an aqueous environment. Aromatic residues such as tryptophan and tyrosine have been shown to be important in the pore region of KcsA (K channel of streptomyces A) channels in determining the final dimensions of the extracellular mouth of the pore [65].

Ideally, the rate of conductance from simulations would be directly relatable to experiments. While we observed multiple ion transport events, there were more ion transport events than WT in most mutated channel simulations, indicating that the length of simulations used may not be sufficient for direct computational measurement of conductance. Future studies will extend our initial work, such as determining the level of simulation details and time required to directly match experimental conductance measurements.

Overall, both the directionality and magnitude of changes seen through computational molecular structural assessment are largely concordant with the electrophysiological studies. This suggests that these assessments are not only useful in clarifying the underlying molecular causes of the measured electrophysiological changes but may also be used alone to provide evidence of pathogenicity. These data enabled the further clinical characterization of KIS and a better understanding of the mechanistic complexity underlying this rare genetic disorder. Currently, ACMGG/AMP guidelines for variant classification have criteria for computational evidence (PP3) and for functional assays (PS3), but the rigor, breadth, and complexity of the computational studies described here fail to be adequately captured by PP3. When cell-based assays are not available for identified variants, the appropriate utilization of computational variant analysis for determining the pathogenicity of an individual variant will become critical and is feasible as herein demonstrated.

This study provides insight into the mechanistic complexity underlying this rare genetic disorder. It has demonstrated that *KCNK9* variants can cause a variety of effects on channel function ranging from upregulating to downregulating current to differing effects on regulation by G_αq_ and extracellular acidification. Yet, the dysregulation of TASK3, whether by an increase or decrease of channel properties, is sufficient to cause a single clinical disorder, KIS, not separable according to the specific functional impact. We may infer, then, that deviations from normal current density in either direction ultimately lead to the same overall physiological effect and clinical outcome. This has been shown in other genetic conditions involving several different potassium channels, including K_V_1.2, K_V_2.1 K_V_7.2, and K_ir_1.2 channels where, paradoxically, both loss and gain of function variants lead to hyperexcitability disorders such as epilepsy [66]. Further, the most consistent observation (for about 70% of the variants) is a loss of regulation. So perhaps, both during development and for functional responses in maturity, the most important observation is not the current size, per se, but the appropriate channel response to regulatory cues. In future work, further insight into the functional consequences of the differing effects of the *KCNK9* variants that we have described could be obtained from iPSC-derived neurons from patients harboring these variants. In addition to studying current through the mutated channels in the neurons themselves, this will allow us to measure the effects of the altered channel function on the excitability and firing patterns of these neurons and will provide a translational model for therapeutic evaluation.

The observed variable effects on channel function caused by the variants in *KCNK9* have further ramifications on potential therapy for KIS. It was previously hypothesized that channel-stimulatory drugs, such as a subset of the nonsteroidal anti-inflammatory fenamic acid class of drugs, may be useful in treating KIS as flufenamic acid had been shown to partially rescue the reduced current in Gly236Arg-mutated channels [14]. Graham et al. described the early results of treatment of two individuals with KIS with mefenamic acid suggesting positive effects [8]. The data presented in this study suggest that at a minimum these medications might only be affective for individuals with a subset of variants causing reduced current, similar to that of Gly236Arg. These data may also suggest caution in treating an individual with normal or increased channel current as we might hypothesize exacerbated detrimental effects. However, it may also indicate that this approach may be challenging to appropriately dose for maximal efficacy or that without achieving proper channel regulation, little benefit may be achieved. An alternative strategy could be to increase expression of the normal paternal allele as demonstrated by Cooper et al. [4]. It is possible that paternal allele expression levels may be contributing to the phenotypic variability observed, and if determined, would support this as a possible treatment approach. It is interesting to hypothesize that clinical benefit may be achieved through treatment with medications such as valproic acid, a known histone deacetylase (HDAC) inhibitor, by promoting paternal allele expression. We observed two patients in our study with seizures treated with this medication showing good seizure control, but further work is needed to determine if other KIS-associated symptoms also show improvement.

## Conclusions

In summary, KIS is a neurodevelopmental disorder caused by a spectrum of pathogenic variants on the maternal allele of the paternally imprinted *KCNK9* gene. Variants disrupt TASK3 channel function by causing gain or loss of current, or current regulation, demonstrating the complexity underlying the mechanism of disease, and likely requiring different methods of treatment. These findings further characterize the clinical features associated with this emerging disorder, demonstrate the utility of computational modeling for understanding variant impact on protein function, and bring caution and suggestions to potential treatment strategies for future studies.

## Supplementary Information

**Additional file 1: Supplementary Note**. Clinical Histories. Written clinical histories including genetic testing for each novel family in this study. Molecular Modeling Reveals Mutation-Specific Effects on Channel Mechanics. Molecular Dynamics Simulations Show Changes in Potassium Ion Distribution.

**Additional file 2: Table S1.** *KCNK9* variants, impact summary, and ACMG classification.

**Additional file 3: Video S1**. Visualization of PC 1, 2, and 3 of the TASK3 dynamic modeling.

**Additional file 4: Video S2**. Video of proband P6.1 demonstrating autistic features with stereotypies.

**Additional file 5: Table S2**. Seizure and seizure-like episodes in affected individuals with at least one afebrile seizure.

**Additional file 6: Figure S1**. TASK3 conformational changes define PC motions and channel gating. **Figure S2**. Detailed view of the TASK3 selectivity filter and surrounding residues. **Figure S3**. Distributions of K+ ions for selected positions along the transport process, and across variants. **Figure S4**. Genomic variants lead to changes in K+ concentration at the selectivity filter. **Figure S5**. Cellular localization of labelled TASK3 variants. **Figure S6**. Comparison of whole cell current density for Tyr205Cys in the presence of various cysteine-modifying agents.

**Additional file 7: Table S3**. A comparison of whole cell current density and reversal potentials between TASK3 clinical variants and matched WT controls.

**Additional file 8: Table S4**. A comparison of inhibition by extracellular acidification (pH 6.4) between TASK3 clinical variants and WT controls.

**Additional file 9: Table S5**. A comparison of GPCRs regulation between TASK3 clinical variants and WT controls.

## Data Availability

All data generated or analyzed during this study we are able to share are included in this published article and its supplementary information files. Raw genetic sequence data are not available as study participants did not consent to sharing their sequence data publicly. Specific questions or requests can be made to the corresponding authors.

## Abbreviations

3-HB: 3-Helix bundle
ACMGG: American College of Medical Genetics and Genomics
ADHD: Attention deficit hyperactivity disorder
AMP: Association for Molecular Pathology
ASD: Autism spectrum disorder
CCR: Constrained coding regions
CI: Confidence intervals
CMR: CellMask Deep Red
CNS: Central nervous system
DTNB: 5″-Dithio-bis(2-nitrobenzoic acid)
DTT: Dithiothreitol
EMG: Electromyography
GFP: Green fluorescent protein
GPCR: G-protein-coupled receptor
HDAC: Histone deacetylase
ID: Intellectual disability
K2P: Two-pore domain potassium channel family
KIS: KCNK9 imprinting syndrome
MD: Molecular dynamics
MTS: Methanethiosulfonates
PCA: Principal component analysis
PCC: Pearson’s correlation coefficient
PDB: Protein Data Bank
RDF: Radial distribution function
RMSD: Root mean-squared deviation
RMSF: Root mean-squared fluctuation
ROC: Receiver operating characteristic
AUC: Area under the curve
STD: Standard deviation
TASK 3: TWIK-related acid-sensitive K channel 3, K2P9.1
TIG: Thr-Ile-Gly motif
TM: Transmembrane
VMD: Visual Molecular Dynamics
VUS: Variant of uncertain significance
WT: Wildtype
