# Supplementary material for: Gain and loss of TASK3 channel function and its regulation by novel variation cause KCNK9 imprinting syndrome
Source: Genome Med. 2022 Jun 13;14:62. doi: 10.1186/s13073-022-01064-4 (PMC9195326; doi:10.1186/s13073-022-01064-4)
Supplement: Supplementary file 1 — Additional file 1: Supplementary Note. Clinical Histories. Written clinical histories including genetic testing for each novel family in this study. Molecular Modeling Reveals Mutation-Specific Effects on Channel Mechanics. Molecular Dynamics Simulations Show Changes in Potassium Ion Distribution. [file 13073_2022_1064_MOESM1_ESM.pdf]

Gain and loss of TASK3 channel function and its regulation by novel variation cause *KCNK9* imprinting syndrome

Additional file 1

Supplementary Note:

Clinical Histories

Family 1: c.391C>A, p.(Arg131Ser)
Probands P1.1 and P1.2 are female full siblings that were confirmed to have a diagnosis of *KCNK9*-imprinting syndrome prior to being seen by the genetics service. Both affected siblings were determined to have a maternally inherited pathogenic variant in *KCNK9*: c.391C>A (p.Arg131Ser).

Proband P1.1 was seen by the genetics service at 2.5 years of age. She was born at 39 weeks gestational age to a gravida 1, para 1 (now G2P2), 24-year-old mother and 24-year-old father via C-section due to cephalopelvic disproportion. Birth weight was 7 pounds (25th percentile) and birth length was 19 inches (25th percentile). Other birth parameters were not available at the time of the evaluation. During the newborn period there were significant feeding difficulties and central hypotonia noted. Prenatal complications included maternal gestational diabetes that was managed by a modified diet. Prenatal diagnostic testing was not elected by the family and all prenatal ultrasounds and maternal serum screening were negative during the pregnancy.

Developmental milestones were delayed, and P1.1 able was able to sit unassisted at 9 months, began crawling at 21 months and walking independently by 23.5 months of age. At the time of the first evaluation, she was nonverbal and has remained so now at 6 years of age. She is able to communicate by gestures and some sign language. She was able to start eating pureed foods around 6 months of age, but solid foods could not be introduced until 2.5 years.

The younger sibling to P1.1 (individual P1.2) was seen for an initial evaluation at 20 months of age. She was born at 39 weeks gestational age to a 26-year-old mother and 26-year-old father via repeat C-section. Birth weight was 7 pounds and birth length was 19 inches, which were both appropriate for gestational age. Prenatal diagnostic testing specifically for the known familial *KCKN9* variant was not elected by the family and all prenatal ultrasounds and maternal serum screening were negative during the pregnancy. The patient was tested immediately after birth with a positive result, and therapeutic services were started via an early intervention program. P1.2 had significant feeding difficulties and is requiring pureed foods still current age of 4 years.

P1.2 sat unassisted at 13-14 months, walked independently at 19 months of age, and was also nonverbal at the time of our first evaluation and has remained so. She is able to communicate by gesture. She exhibits further comprehension compared to her sister but is unable to follow complex instructions. There is a question as to whether she may have had absence seizures.

Both children have evidence of hyperactivity, loud verbalizations, and have some other behavioral issues including trichotillomania, which was managed by redirection of behavior. P1.1 has required melatonin due to fragmented sleep patterns. P1.2 has skin sensitivity to sunlight, often developing a pruritic red papular rash that occurs within 2-3 hours of exposure akin to polymorphic

light eruption. Allergy testing was unremarkable. P1.2 also has a formal diagnosis of ADHD and is on some pharmacotherapy and both are noted to have aggressive behavior. Both children have also been receiving supportive speech and occupational therapies.

Probands P1.1 and P1.2 are the only children for this parental union. Family history was unremarkable for other individuals with similar features. Parental ancestries are mixed European and there was no history of consanguinity.

On exam, P1.1 exhibited normal growth parameters for age and pertinent findings included minor dysmorphic features (arched eyebrows that are flared medially, tented upper lip with myopathic appearing facies), normal appearing hands and feet with slightly aberrant palmar creases. The patient normally requires AFOS. On neurologic exam, the patient was nonverbal and there was relatively good muscle tone with noted brisk patellar reflexes. When AFOs were removed the patient was able to easily bear weight and was running and climbing although significant joint hypermobility was noted at the knees bilaterally.

Physical examination, P1.2 also exhibited normal growth parameters for age with minimal facial dysmorphism which mainly consisted of a slightly tented upper lip and myopathic facies. The remainder of the examination was unremarkable, although on neurologic evaluation there was central hypotonia with +1 Achilles tendon reflexes.

A clinical next-generation sequencing multigene panel test identified the c.391C>A, p.(Arg131Ser) *KCNK9* variant in proband 1.1 and targeted Sanger sequencing revealed this variant is present in the affected sister (P1.2).

Family 2: c.391C>T, p.(Arg131Cys)

Proband 2.1 is the first child of healthy parents with unremarkable family history for syndromic disorders and intellectual disability. He is male and was born at term after an uneventful pregnancy with a weight of 3235 g (-0.95 SD), a length of 55.0 cm (+1.01 SD) and a head circumference of 35.0cm (-0.53 SD). After birth, he presented with muscular hypotonia, feeding difficulties in the first weeks of life and apneic episodes, which were treated with caffeine until the age of four months. A routinely conducted EEG showed spike waves without clinical symptoms. Metabolic screening revealed a positive sulfite test. At the age of two months, control of EEG was normal and further metabolic testing was unremarkable. Creatine kinase value was normal.

At the age of twelve months he was able to sit independently. Walking independently was possible at the age of 16 months but he still presented planovalgus feet. Neuropediatric assessment at the age of eleven months confirmed proximally emphasized muscular hypotonia which was markedly improved at the age of 16 months. Proprioceptive reflexes were normal. At age 19 month, he was able to speak syllables but did not use full words. The parents did not note abnormal behaviour.

External conducted genetic testing for Prader-Willi syndrome and spinal muscular atrophy was negative. Further genetic diagnostics was initiated from the neuropediatric department at the age of eight months and remained negative for clinically relevant variants.

Proband P2.1 was last reviewed at the age of 19 months. His height was 81.5 cm (-0.63 SD) and weight was 10.8 kg (-0.54 SD). His head circumference was 47.0 cm (-1.38 SD). Dolichocephaly and signs of myopathic facial expression as well as a subtle sacral dimple were observed during physical examination.

A research-based trio exome analysis was pursued. The de novo missense variant NM_001282534.1: c.391C>T, p.(Arg131Cys) in *KCNK9* (chr8[hg19]: g.140631235G>A) was identified by exome sequencing. Paternity and maternity was confirmed.

Family 3: c.392G>A, p.(Arg131His)

Proband 3.1 is a 12-year-old Hispanic boy with borderline cognition, mixed language disorder, developmental delay, short stature, resolved congenital hypotonia, disruptive behavior disorder, and abnormal brain MRI with delayed myelination. He was born preterm at 36 weeks to non-consanguineous parents via C-section due to non-reassuring fetal monitor. Hypotonia was noticed in the neonatal period. Motor development was delayed. He could sit unsupported and started to crawl at 18 months and walked at 25 months. He had his first word at 2 years with a significant speech delay later on. At age 6 he could use only 30-40 words in 2-3 words sentences. At the age of 10, he could talk in full sentences. No history of feeding difficulties in early childhood but had difficulties in swallowing solid foods with frequent chocking. He could eat table food only at 2 years of age. He had a normal swallow test. He has behavioral abnormalities. His mother reports that in early childhood he expressed no feelings. He did not cry during early childhood, not even after immunizations shots with possible low sensitivity for pain. At later age he started to complain of pain and developed a friendly and "bubbly" personality with happy demeanor. He is attending a Life Skills program at school. On a formal developmental evaluation, he was found to have borderline intelligence and was defined as "slow learner" yet not qualified to the diagnosis of "intellectual disability". He was also found to have mixed language disorder and disruptive behavioral disorder. He had single febrile seizure at around 15 months of age with no further seizures. He has normal Echo and EKG. He has vision refractive error and nearsightedness. No hearing concerns.

Brain MRI showed delayed myelination involving the subcortical U fibers of the frontal and parietal lobes. Repeat MRI showed mild improvement with increased age. There were continued foci of increased T2 and FLAIR signal perhaps representing small foci of hypomyelination or other causes for incomplete myelination perhaps are merely related to perivascular spaces. No progression and no new lesions were seen.

Proband P3.1 was found to have a *de novo* missense variant of uncertain significance (VUS) in *KCNK9* (c.392 G>A, p.(Arg131His)) on a proband exome sequencing. He also has heteroplasmic pathogenic variant in the mitochondrial genome m.3250T>C (tRNA >Leu), with 21.5% heteroplasmy.

Family history is positive for a mother and three sisters with the mitochondrial change (*MT-TL1* m.3250T>C) with 10-20% heteroplasmy. His mother has congenital microphthalmia and unilateral blindness and was also found to have 10-20% heteroplasmy (*MT-TL1* m.3250T>C). She complains of fatigue and muscle cramps. Maternal GM died sudden death at the age of 64. She had hearing loss that was diagnosed in her 40s. She also had diabetes mellitus, hypertension, and ischemic heart disease.

On physical exam at the age of 10 years P3.1 was found to have short stature with height Z score of -2.26 and weight with Z score of -2.17 (according to CDC growth charts). His head circumference is at the 25th centile. He had dysmorphic facial features including dolichocephaly and bitemporal narrowing with low set ears and big mouth. Palate intact. His hands are small (less than 3rd centile for age) with no deformity.

Previous diagnostic testing included: CMA (CMA-HR+SNP(V9.1.1) - copy number gain of chromosome band Xp22.31 in the Steroid Sulfatase (STS) region of approximately 1.249 Mb in size (is not thought to explain his clinical findings). Methylation for AS/PWS - normal. Fragile X - normal. Normal lactate, plasma amino acid, acylcarnitine profile, urine organic acids, and muscle enzymes. Clinical trio exome sequencing was pursued through Baylor Genetics (10/15/13) revealing 21% heteroplasmy of the *MT-TL1* pathogenic variant (m.3250T>C) and the *de novo* c.392G>A; p.(Arg131His) variant in *KCNK9*.

Family 4: c.392G>A, p.(Arg131His)
Proband P4.1 is a 5 year-old Indian female with global developmental delay, central hypotonia, and a single febrile seizure. After a normal delivery she was noted to be skinny, with very little body fat. Parents were concerned about her from birth because she never cried. She had a poor suck and would only bottle feed. At 10 months she presented with hypotonia and poor head control. As an infant, mild hypotelorism was noted but otherwise she was nondysmorphic. She had a single febrile convulsion at 1.3 years. At five years of age she follows some commands, and speaks in phrases. She has slight hypotonia in arms and normal tone in legs. There is no spastic catch but she has a mild head lag. Her gait is normal. She is in pre-school and with therapy has been making steady progress with no regression. An MRI at 11 months was normal. CMA testing and methylation PCR for Prader Willi and Angelman Syndrome were negative. Clinical exome sequencing through GeneDx (Gaithersburg, MD) of proband, mother, and father showed a heterozygous *de novo* missense variant (c.392G>A; p.(Arg131His)) in *KCNK9* (NM_001282534.1). She was also found to be heterozygous *de novo* and mosaic for the variant c.1016G>A; p.(Gly339Glu) in *PLCB3* (NM_000932.2). She had a homozygous deletion of exons 5-16 of *SLC18A1* (NM_003053.3) which were inherited from both parents. Neither *PLCB3* or *SLC18A1* have a known Mendelian disease association to date.

Family 5: c.392G>A, p.(Arg131His)
Proband P5.1 is a 6-year-old female with a history of global developmental delay, intellectual disability, attention deficit disorder, hypotonia and cleft palate. This individual's developmental delays were first noted at ~9 months of age. Gross motor and speech domains are most severely affected. Currently, she has significant issues with attention deficit, hyperactivity, and impulsivity. She also seems to have decreased pain sensation. Phenotypically, she is nondysmorphic. Imaging studies including brain and total spine MRIs have been normal. An EEG was performed related to starring spells and was also normal. The patient has not had seizures. Surgical history includes tonsillectomy, adenoidectomy and PE tube placement

Whole exome sequencing through Ambry Genetics (Aliso Viejo, CA) identified a *de novo* likely pathogenic variant in *KCNK9* (c.392G>A; p.(Arg131His)). Parent of origin for the allele carrying the *KCNK9* variant has not been determined. Additional genetic tests including karyotype, CMA, and fragile X were normal. Metabolic studies were also normal.

Family 6: c.392G>A, p.(Arg131His)
Proband P6.1 is an 18-year-old female. She was born post-term by spontaneous delivery after an uneventful pregnancy from unrelated parents of Caucasian origin. Family history was also unremarkable.

Her birth parameters were all in the normal range (weight 3.050kg, length 51cm, OFC 34cm, APGAR score 9/10). Neonatal period had been characterized by axial hypotonia (neck, trunk,

shoulder girdle) and feeding difficulties (poor sucking and poor appetite), she also presented wrist in flexion and bilateral club foot for which surgery was performed at four months.

Her psychomotor development was regular until her first year of life (sitting position 6 months, ambulation 14 months), when an arrest and regression of social and language skills was noted. She also began to show slowly worsening behavioral problems (autistic features, hands stereotypies, attention disorder and oppositional disorder). Please see **Additional file 4: Video S1**.

At the pediatric evaluation, diffuse muscle hypotrophy prevalent in the lower limbs (right greater than left) and joint laxity were observed. Heart and abdominal ultrasounds, EEG and brain MRI were normal. No audiological or ophthalmological abnormalities were observed. She never experienced seizures.

She was referred for the first dysmorphology evaluation at about 9 years and 5 months of age. Height was 131 cm (25th centile), weight 23.5 kg (3rd centile), OFC was 50 cm (3rd-10th centile). Peculiar facial dysmorphisms (arched eyebrows, slight synophrys, broad and short philtrum, thick and everted upper and lower lips, open mouth with thick alveolar ridges and prominent incisors overbite) and mild generalized hypertrichosis were observed.

At 15 years, height was 153 cm (10th -25th centile), weight 35 kg (<3rd centile), OFC was 53 cm (10th -25th centile), she showed some motor stereotypies, like hand clapping or “beating her right foot”. She had a severe speech disorder affecting both articulation and language production.
Behaviorally, she became increasingly irritable, she began throwing objects and showing episodes of self-harm. She started having frequent fear attacks caused by unexpected noises, particular objects or with no apparent causes.

Prior uninformative genetic testing included blood karyotype, array-CGH, *FMR1* testing, sequence analysis and MLPA of *TCF4*, *MeCP2* and *FOXG1*, and methylation analysis of *SNRPN* in the PWS/AS critical region. Targeted NGS with a gene panel for intellectual disability (1) revealed rare missense variants in *SLC6A1* (NM_003042: exon12: c.1250G>A: p.(Arg417His)) and *RELN* (NM_005045: p.(Met1263Ile)) genes, both resulted to be maternally inherited and thought non-contributory. Exome sequencing was pursued at University-Hospital of Padova, Italy analysis, and identified the heterozygous, *de novo*, c.392G>A (p.Arg131His) VUS in *KCNK9*, previously reported as likely pathogenic. An allele-specific PCR analysis demonstrated that the variant arose on the maternal allele.

Family 7: c.392G>A p.(Arg131His)
Proband P7.1 was seen at the age of 8 years in medical genetics clinics for intellectual disability. He had no family history of neurological or neurodevelopmental disease. He came from a spontaneous pregnancy and was born at 31 weeks of gestation secondary to an IUGR. Birth size: 46.5cm (50th percentile), weight 2.120 kg (50th percentile), head circumference 46.5 cm (50th percentile). Neonatal period was characterized by hypotonia and feeding difficulties. He was able to sit at the age of 12 months and to walk at 21 months. He had delayed language. He is currently in a specialized school. He is able to recognize some words. He also presents some issues with aggressive behavior. No history of epilepsy.

At the age of 8y, he weighed 33kg (90th percentile), his size was 1,33m (+1DS), his head circumference was 53cm (+0.5DS). He had a normal neurological exam. He presents with some facial dysmorphism including thin upper lip, open mouth, arched eyebrow, and everted lower lip).

Karyotype, Fragile-X expansion, SNP-array were normal. Trio-based exome sequencing (2) identified a *de novo* missense variant (c.392G>A p.(Arg131His)) in *KCNK9*.

Family 8: c.392G>C, p.(Arg131Pro)
Proband 8.1 is female and was born to a 31-year-old gravida 1 para 1 Puerto Rican woman and 28-year-old father after an uncomplicated pregnancy. No maternal infections, diabetes, hypertension were noted. Amoxicillin was taken for dental work, but no other medications. No reported smoking, alcohol or drug use. Ultrasound at 20 weeks noted bilateral club feet and referred was made to Perinatal Center. No amniocentesis was performed. Normal fetal activity was detected. Transferred to NICU w/multiple congenital anomalies. In NICU for 27 days.
She was referred for genetics consultation and seen in clinic at 6 weeks of age. She was having feeding problems and metopic and sagittal ridges were apparent. At that time there was growth delay (weight, length and head circumference all below 1st percentile). Dysmorphic features included down-slanting palpebral fissures, midline posterior cleft palate, micrognathia, glossoptosis, redundant nuchal skin, hypertrophic clitoris, apparently short limbs, distal arthrogryposis (hands, ankles), and generalized hypotonia with weak cry.

Developmental milestone delays ensued and she had normal karyotype (46,XX). SNP microarray was normal. Skeletal survey revealed scoliosis and mesomelia without dysplasia. CT head revealed that the sutures were overlapping but actually open (not craniosynostosis as had been the clinical impression). Brain MRI revealed ventriculomegaly. Clinical trio exome sequencing through Ambry Genetics (Aliso Viejo, CA) revealed the c.392G>C; p.(Arg131Pro) variant in *KCNK9*.

Family 9: c.395T>G, p.(Met132Arg)
Proband P9.1 was born following normal pregnancy, erratic prolonged labor, born in poor condition, flat, floppy and cyanosed. He had hypotonia, weak cry, and failed to straighten his legs for the first 2 weeks. He has global developmental delay and sat at 9 months, crawled at 13 months, and walked at 15 months. He has had recurrent respiratory tract infections until the age of 5, and has been non-verbal for a very long time, now simple indistinct speech. He lives in a care facility and still has significant behavior problems at 26 years of age with profound intellectual disability.

The younger maternal half-brother (P9.2) was born following a normal pregnancy. He had increased nuchal translucency antenatally and born by emergency C-section for heart rate decelerations. He had a high-pitched cry as a baby and was generally placid as a child. Recurrent respiratory tract infections until 5yrs of age were noted. He has mild developmental delay and sat unsupported at 8 months, crawled at 11 months, walked at 15 months. He had ~10 single words and at 14 months stopped talking and autistic traits started to emerge. Echolalia initially, now cannot speak. P9.2 attends special needs school, has moderate intellectual disability, and is unable to live independently. At 21 years of age, he is tall and slim with a marfanoid-like appearance and joint hypermobility.

Genetic testing was pursued through the Genomics England 100,000 Genomes Project whereby targeted molecular analysis of genes restricted to the intellectual disability panel (version 1.673) was undertaken identifying the maternally inherited c.395T>G, p.(Met132Arg) *KCNK9* variant in both affected siblings (P9.1 & P9.2).

Family 10: c.403_405delTTC, p.(Phe135del)
Proband P10.1 is an African American female who was referred to genetics at age 5 months for evaluation of hypotonia and is currently 4 years 6 months old. She initially presented at 48 hours after birth with profound encephalopathy with poor reactivity but had a normal EEG and brain MRI. She later started exhibiting developmental delay. She is followed by neurology for axial and appendicular hypotonia as well as expressive more than receptive speech delay. Ophthalmological evaluation revealed mild hyperopic astigmatism OU, benign end gaze nystagmus OU but no strabismus observed. Her fundoscopic exam was normal. She exhibits mild right-side thoracolumbar scoliosis (17 degrees) with mild hip dysplasia. She was prescribed AFO's to support muscle tone in her lower limbs.

She started walking at 2.5 years; currently, walks tippy toes, can run and ride a tricycle. She is putting sentences together. She has more than 200 words. She follows directions. She is very social. She can dress and undress herself. She can throw and catch a ball but not consistently due to her hypotonia. She has some coordination issues. She struggles with a few fine motor skills and needs help putting on her shoes. She is fully toilet trained. She attends pre-K and has an IEP in place. She receives physical, occupation and speech therapies.

On her physical exam, her weight is at the 14th percentile (Z= -1.10), height at the 32nd percentile (Z= -0.48) and OFC at the 45th percentile (Z= -0.12). She exhibits dysmorphic features and thin body habitus. She has long face, blue sclerae, long eyelashes, low set and posteriorly rotated ears, high arched palate, upturned nares, short philtrum, tented upper lip, opened mouth in resting position, and hypotonic myopathic-like face. Her skeletal findings include long 1st and 2nd toes bilaterally as well significantly pronated and flat feet bilaterally. She exhibits generalized hypotonia with symmetric movement of extremities.

Clinical exome sequencing was pursued through GeneDx (Gaithersburg, MD) and revealed a de novo c.403_405delTTC, p.Phe135del variant in *KCNK9*.

Family 11: c.466A>G, p.(Met156Val)
Proband P11.1 is male and was the first child of a non-consanguineous couple. Pregnancy was uneventful. He was born at 38 weeks' gestation (OFC 36 cm, weight 3420g, height 52cm).

He walked at 18 months old, with mild global motor delay. He also had moderate speech delay with good evolution. At 11 years old, he had moderate learning difficulties associated with relational difficulties and troubles with abstraction with fine motor trouble and slowness, but normal school and mild stereotypic features, aggressiveness, anxiety with bruxism, attention disorder with hyperactivity. He had febrile episode of status epilepticus at 3½ years, and non-febrile tonic seizures at 8 years old well controlled by lamotrigine. EEG demonstrated spike and wave discharges at 8 years of age. He was also treated by Melatonin for sleep difficulties. Brain MRI was normal.

Clinical examination at 11 years old showed very mild hypotonia, mild non-cerebellar tremor, flat feet, and short hands, (OFC 56 cm (+2 SD), Weight 36 Kg (+1.5 SD), Height 150 cm (+1 SD)).

Genetic explorations were done to explore psychomotor delay, seizures, and behavioral abnormalities. Array CGH was normal, X-Fragile was ruled out. Clinical solo exome sequencing through the University Hospital Nantes (Nantes, France) identified the heterozygous c.466A>G, p.Met156Val variant in *KCNK9*, classified as a VUS. Family studies by Sanger sequencing showed that the healthy mother and the healthy maternal grandfather carried the same variant.

Family 12: c.477G>A, p.(Met159Ile)

Proband P12.1 is male and the third child born at 41 weeks gestation to a 35-year-old at term after spontaneous labor following an uncomplicated pregnancy. His two older siblings are reportedly healthy. The mother also had a medical termination of pregnancy for hydrops without etiology despite medical investigations (46 XX karyotype, no macroscopic malformation, panel of storage disorder negative). No relevant familial history or consanguinity was identified. His Apgar scores were 10 at 1 minute, 5 and 10 minutes with cord PH 7.28. He presented a good adaptation to extrauterine life but no cry. His birth weight was 3090g (9th percentile), body length 49 cm (8th percentile), and head circumference 34 cm (17th percentile).

Examination on the first day found the child to be difficult to wake, with an axial hypotonia and peripheral hypertonia. Osteotendinous reflexes were present but archaic reflexes were uncompleted (no stepping, moro and rooting reflexes but a weak suck reflex). Morphologic examination found reducible club hand and foot and ogival palate. Infectious hypothesis was eliminated after bacteriologic testing (cerebrospinal liquid and blood culture). In addition to global hypotonia from birth, there was paroxysmal truncal and axial limb hypertonia with hyperkinetic movements from 3 months to 9 months, poor eye contact and strabismus, and at 1 year, truncal hypotonia with hyperlaxity of four extremities and knees. Initial neurologic (electroencephalogram, cerebral RMI, muscular biopsy normal, fundus examination), genetic (CGH-array, Prader-Willi, Steinert and spinal muscular atrophy) and metabolic (urinary organic acid chromatography, plasmatic and urinary amino-acid chromatography, CDG syndrome) investigations were normal.

Developmentally, he achieved head holding at 1 year, rolling over from his back onto his stomach and from his stomach onto his back at 15 months, sitting at 18 months, and crawling at 2 years of age. With regard to interaction, as an infant, he interacted with those around him. He pointed his finger at objects that interested him, laughed, and cried. Hypotonia persisted with scoliotic posture which required corset. Seizures appeared at 26 months and required bi-therapy by sodium valproate and lamotrigine.

Trio-based exome sequencing (2) identified the de novo c.477G>A, p.Met159Ile variant in *KCNK9*.

Family 13: c.477G>A, p.(Met159Ile)

Proband P13.1 is an 8-year-old boy, who came to the referral center consult at the age of 34 months due to suspicion of neuromuscular disorder.

He was born at term by C-section due to failure to progress, with APGAR scores of 7 at 1 min and 10 at 5 min, from non-consanguineous parents with no siblings. Since birth, he has had axial and peripheral hypotonia, poor sucking reflex, dysphagia, and failure to gain weight. At day 6 of life, he developed respiratory distress and was put on transient non-invasive ventilation. At the age of 42 days, he presented with spasms (myoclonic) and EEG showed intermittent pseudo periodic pattern and no epileptic discharges. At the age of 2 years and 4 months, he had several brief episodes of tonic limb flexion associated with gaze fixation. EEG performed showed generalized spikes and spike waves so he was put on levetiracetam, which was given for few months but stopped by the mother due to reportedly no changes in seizures frequency.

At 2 years and 10 months of age, he showed poor gaze fixation, eyelid ptosis, facial and limb muscle weakness, reduced spontaneous and touched-evoked movements, and severe axial hypotonia. He has cognitive delay and failure to thrive.

Diagnostic work-up performed showed no abnormalities in: brain MRI at day 9 and 40, cardiac exam (ECG, ultrasound), metabolic workup (including level of maltase acid, test for congenital disorders of glycosylation), thyroid function, lipid profile, auditory evoked potential, ophthalmologic exam, and dexascan. Genetic initial testing included karyotype, CGH array, search for Steinert's disease, Prader-Willi, Noonan, and Smith-Lemli-Opitz syndromes, and *SMARCA2* were negative. Neuromuscular workup included: normal CK and muscle biopsy at 1 year showed predominant type 2 fibers. Repeated at age of 4 years showed major atrophy of type 2 fibers, persistence of an important number of undifferentiated muscle fibers. Whole body MRI at the age of 4 years showed diffuse muscle atrophy, with no fatty infiltration. Electrodiagnostic study at 14 months showed normal nerve conduction and normal EMG. Exam repeated at the age of 3 years showed decreased CMAPs, normal nerve sensory and motor nerve conduction studies, slow repetitive stimulation testing (2Hz) no decrement, high frequency repetitive stimulation testing showed positive increment which is suggestive of abnormalities in the presynaptic neuromuscular junction that is seen in Lambert Eaton. Further analysis related to Lambert Eaton syndrome ruled-out paraneoplasia, and he was negative for anti-MUSK, anti-ACH, and anti-calcium channel antibodies. Genetic sequencing for *CHAT* was negative. Clinical NGS of 4800 genes using the Truesight one sequencing panel (Illumina) through the Laboratoire de Biochimie et de Biologie Moléculaire (Central Hospital University, Nancy, France) revealed the *de novo* variant, c.477G>A, p.(Met159Ile) in *KCNK9*.

Proband P13.1 developed refractory epilepsy. During follow up, he had suspicion of gaze fixation so EEG was done at the age of 3 years and showed focal epileptic discharges therefore he was treated with valproate. Shortly after, he developed a status epilepticus during hospitalization and EEG was compatible with Lennox-Gastaut syndrome so treatment was modified and seizures were controlled under triple therapy valproate, lamotrigine, and clonazepam. Interestingly on these antiepileptic drugs there was a slight improvement of his tonus so EMG was controlled and showed at 3 years and 10 months of age normalization of CMAPs, no decrement on slow repetitive stimulation testing (2Hz), and 70% increment on high frequency repetitive stimulation testing compared to 278% on the initial testing without antiepileptic drugs. Trial of treatment for suspected congenital myasthenia syndrome failed to show any improvement on pyridostigmine nor salbutamol. 3,4-diaminopryridine was not prescribed due to his seizures. Polysomnography as part of his neuromuscular workup showed repetitive desaturation and hypercapnia so NVI was prescribed.

At the age of 7 years, he has severe neurodevelopmental delay with no improvement since age of 4 years (no language, can fix and follow with his eyes, turn to vocal stimuli, can grasp objects with both hands and throw them). He still has feeding difficulties and can swallow only mixed food. Failure to thrive, bilateral fluctuating ptosis, diffuse muscular atrophy, axial and peripheral hypotonia, if put on sitting position he can sit alone, thoracolumbar scoliosis (for which he has Garchois bracing), contractures at both ankles (treated with orthesis).

Family 14: c.487G>C, p.(Gly163Arg)

Proband 14.1 is a 16-year-old male who is the youngest child of three children born to healthy unrelated German parents. He was born by caesarian section at 38 weeks of gestation with low weight (2590g; 3rd centile), normal length (48cm; 25th centile) and normal head circumference (34cm; 25th centile). Immediately after birth, he showed muscular hypotonia, temperature instability, bradycardia and irregular breathing pattern. EEG, EKG and echocardiogram as well as screening for vision and hearing were unremarkable. Initial genetic testing including SNParray, conventional karyotyping and *SMN1* copy-number and sequence variant analysis as well as screening for congenital metabolic disorders and congenital disorders of glycosylation yielded

negative results. Cerebral MRI at the age of 2 years presented a slight enlargement of cerebrospinal fluid space prominent around the Sylvian fissure region. There were no signs of delayed myelination, organic malformations or joint laxity. Otherwise, his medical and family history was unremarkable. After the EEG examinations were inconspicuous in infancy and childhood, pathological EEGs with isolated sharp waves have appeared in recent years, but seizures have not occurred so far. At the age of 15 years, he exhibited severely delayed psychomotor development with facial dysmorphism (high palate with prominent teeth, upslanting palpebral fissures and large, low-set ears) and muscular hypotonia. He had not learned to speak words and therefore communicated with hand gestures. He could only walk and eat with help. His measurements were in the normal range (height: 164cm, 50th centile; weight: 45kg, 10th centile; head circumference: 53cm, 3-10th centile). Trio exome sequencing revealed a maternally inherited *KCNK9* missense VUS (c.487G>C, p.(Gly163Arg)) in a heterozygous state. Sanger sequencing confirmed the *KCNK9* variant in the proband and his mother, whereas his maternal grandmother, his father, and his healthy brother and sister were shown to carry the wildtype allele. Maternal grandfather was not available. Segregation analysis for a benign SNV (rs2615374 C>T) in exon 2 of *KCNK9* showed that the mother is a heterozygous carrier (C/T) of this variant, while the affected son and his father are homozygous carriers of the C allele. The maternal grandmother is also heterozygous carrier of this variant. Together with the findings of the siblings, who are both heterozygous for the SNV, this allows the conclusion that the variant c.487G>C is located on the mother's paternal allele, either inherited from her father or occurred *de novo* in her.

Family 15: c.491T>G, p.(Phe164Cys)

Proband P15.1 is male and born after an uneventful pregnancy, at a gestational age of 38+2 weeks. Birthweight was 3050 grams. He was a quiet baby. His motor milestones were slightly delayed; he could walk unaided at the age of 19 months. He developed no speech and was diagnosed with an autism spectrum disorder and moderate intellectual disability. Hearing was normal and vison was normal with correction for hypermyopia and astigmatism. He is the fourth of seven children. One of his sisters died in childhood of leukemia. Both parents are deceased.

At the age of 61 he has good general health and lives in a sheltered environment. He developed a compulsive disorder, for which he receives medication (clozapine). At physical examination his height is 182 cm (-0.3 SDs), weight 72.4 kg (BMI 21.9) and head circumference 59.2 cm (0.8 SDs). No dysmorphic features were noted.

SNP-array and *FMR1* analysis revealed no abnormalities. Clinical exome sequencing at GenomeScan and Laboratory for Diagnostic Genome Analysis (Leiden, Netherlands) showed a heterozygous variant (c.491T>G; p.(Phe164Cys)) in *KCNK9*. This variant was tested in the father in DNA isolated from stored colon tissue and could not be demonstrated. Unfortunately, analysis of the variant in the mother was not possible suggesting either maternal inheritance or *de novo* occurrence.

Family 16: c.595A>G, p.(Thr199Ala)

Proband P16.1 is female and was born after an uneventful pregnancy, at a gestational age of 40+1 weeks. Birthweight was 3050 grams, head circumference 34.5 cm. In the first months, parents noted that contact was different. Motor development was normal (walking unaided at the age of 17 months), but speech was delayed. She attended a school for special education. She was diagnosed with ADHD, hypotonia, and moderate intellectual disability. Hearing was normal, vision was normal after successful treatment for amblyopia. She is the second of three children; her siblings are healthy. Mother died of dyskeratosis congenita, which runs in her family.

At the age of 20 years she is in good health. She lives in a sheltered environment, and is able to do simple packing work. She has a reasonable vocabulary. At physical examination she has a hypotonic posture, ptosis, mild hypertelorism, a rather large nose and mild retrognathia. Her height is 176 cm (0.9 SD), weight 50.5 kg (BMI 16.3) and head circumference 57.5 cm (1.3 SD).

Prior testing included karyotyping, *FMR1* analysis, metabolic analysis, MRI of the brain and SNP-array and revealed no abnormalities. Analysis of *DMPK* showed no repeat expansion. Clinical exome sequencing at GenomeScan and Laboratory for Diagnostic Genome Analysis (Leiden, Netherlands) demonstrated a maternally inherited heterozygous variant (c.595A>G: p.(Thr199Ala)) in *KCNK9*.

Family 17: c.614A>G, p.(Tyr205Cys)

Proband P17.1 is male (current age of 18 years) with a clinical history (at last follow up) including a cheerful demeanor, moderate intellectual disability, ADHD, paroxysmal vertigo, and short stature. He has a paternal history of depression, short stature, and learning challenges and a maternal history of learning challenges.

Prior investigations included: brain MRI brain demonstrating non-specific foci of increased T2 signal in the deep white matter, normal ECHO, and normal ECG. Unrevealing genetic testing included karyotype, chromosomal microarray, and subtelomeric FISH.

Trio-based exome sequencing was pursued as part of CAUSES Study (University of British Columbia, institutional review board study number: H15-00092) and identified the maternally inherited c.614A>G, p.(Tyr205Cys) variant in *KCNK9*. The variant was verified as being present in the proband and mother, and absent in the father, with Sanger sequencing by the Genomic Diagnostics Laboratory of British Columbia Children's Hospital (Vancouver, BC).

Family 18: c.706G>C, p.(Gly236Arg)

The clinical history for P18.1 was previously described (3), but he has since been diagnosed with seizures.

At 1 month of age, the proband was noted to have episodes of abdominal crunching and back arching which had a temporal relationship to feeds. He was evaluated by Neurology at 2 months of age and these events were thought to be related to reflux. An EEG around 3-4 months of age showed slowing and some focal sharps which were thought to be normal for patient's age. A repeat EEG at 8 months of age was normal.

At 15 months of age, he had an episode concerning for seizure like activity. The episode was described as acute onset of irritability with abnormal eye movement to one side and has arrhythmic twitching movements all over the body with no associated loss of consciousness. This event lasted for several seconds and was followed by fatigue. A repeat EEG at this time showed slowing in right posterior regions. No epileptiform discharges were seen. He was not started on antiseizure medications.

At 5 years 11 month of age, he started having new events concerning for seizures. The events were characterized by left arm raised up and shaking, with eyes staring and deviated to left, followed by confusion and falling asleep. The event lasts less than 30 seconds. Has had 9 total seizures when Trileptal was started. Trileptal provided good seizure control. Poor sleep was

noticed by family as a trigger for break through seizures. An EEG was repeated after this event which revealed multifocal spike discharges with atypical generalized spike waves and bitemporal slowing and generalized paroxysmal fast activity with maximal impact at right temporo-parieto-occipital area.

Family 19: c.706G>C, p.(Gly236Arg)

The Clinical history of proband P19.1 was previously described (3). Current evaluation revealed that the oropharyngeal dysphagia requiring nasogastric tube feedings improved with therapy and the nasogastric tube was removed at 3 years of age. She is now able to feed well and takes food of different textures. At 15 months, she babbled mainly vowels. She developed a few single words at 18 months of age and spoke in 2-3 word phrases at 2 years 7 months old. Her fine motor skills were initially noted to be delayed, but improved greatly by 2 years 7 months old where she was able to scribble and stack blocks. During her last review at 5 years 8 months old, she was noted to be slightly hypotonic and had poor balance as compared to her peers. She was able to jump and hop on both legs, but still required help in going down stairs. She was able to converse well, but had reduced intelligibility. On the WPPSI-IV, she obtained a Full Scale IQ (FSIQ) of 70. She scored in the extremely low range for working memory and in the borderline range for visual spatial and verbal comprehension. She scored in the low average range for processing speed and in the average range for fluid reasoning. She was also noted to have features of Attention deficit hyperactivity disorder.

Family 23: c.706G>A, p.(Gly236Arg)

Proband P23.1 is male and was born by C-section to a G2P0010, 30 year old mother at 38 weeks' gestation. The pregnancy history was significant for gestational diabetes which was controlled by diet. A third trimester sonogram revealed polyhydramnios. After birth, patient spent 2 weeks in the NICU due to feeding problems. He was thought to have a submucous cleft palate and Pierre Robin sequence, and received NG feedings until 3 days prior to his discharge.

He was evaluated through early intervention services at 2 months of age due to continued feeding problems and hypotonia. He began receiving feeding therapy, OT and PT at 3 months. He rolled at 6 months, could hold a sit at 9 months, and was able to get into a sitting position on his own at 12 months. He was evaluated in Medical Genetics at 15 months of age. On exam, he was noted to have moderate-severe micrognathia, submucous cleft palate with bifid uvula, global developmental delays, and diffuse axial hypotonia with hip/ankle tightness. A previous workup, including plasma amino acids, urine organic acids, acylcarnitine profile carbohydrate deficient transferrin and a SNP microarray was normal. Very long chain fatty acids, Prader-Willi methylation studies and total/free carnitine were performed and were normal. A skeletal survey showed bilateral hind foot valgus, bilateral coxa valgus with shallow acetabulum, and mild uncovering of the femoral epiphysis. His bone age was delayed (bone age of 3 months at a chronological age of 15 months). At 21 months of age, clinical exome sequencing (GeneDx; Gaithersburg, MD) revealed a *de novo* c.706G>C; p.(Gly236Arg) variant in *KCNK9*. Patient was started on 50 mg 3X/day of mefenamic acid at 22 months. This was advanced to 100 mg 3X/day at 24 months. He eventually walked at 28 months of age. He was last seen in Medical Genetics at 3 years and 8 months of age. At that time, he was in a 9:1:3 preschool class where he was receiving PT, OT, and ST. He was able to run and kick a ball. He was doing simple puzzles and knew some letters. He had no words but was able to communicate using some signs. He shakes his head for yes and no and can follow commands. He has had a bloody stool and has had frequent nosebleeds, but has continued on mefenamic acid without other problems. His physical examination is unchanged.

Family 24: c.709G>T, p.(Ala237Ser)
Proband 24.1 is a 6-year-old male that was born to healthy unrelated German parents after a normal pregnancy at 40 weeks of gestation with normal weight (3960 g; 50th centile), length (52cm; 10th centile) and head circumference (36 cm). Starting from the age of 6 months on he received physiotherapy due to muscular hypotonia and delayed motor development. Other laboratory testing was unremarkable. He was able to turn at 8 months, sit at 12 months, crawl at 13 months and walk at 16 months of age. At the age of 2 years, he spoke simple words as “mama” and “papa” and had good language understanding and could follow instructions quite well. However, his language development was severely impaired and his speech did not develop any further. Neurological examination was normal apart from muscular hypotonia. He did not show any dysmorphic features. EEG recordings, EKG, echocardiogram and sonography of the brain were unremarkable. He did not have any organic malformations or complicated hospitalizations. His younger 4-year-old sister, who initially showed normal development, apparently started to show mild language delay. Previous genetic testing including array CGH and conventional karyotyping were normal. Trio exome sequencing and subsequent carrier testing identified a maternally inherited missense variant (c.709G>T, p.(Ala237Ser)) in both siblings with the maternal healthy grandfather being a heterozygous carrier.

Family 26: c.710C>A, p.(Ala237Asp)
Proband P26.1 if male and was born as the first child of healthy non-consanguineous parents (with unremarkable family history concerning genetic and neuromuscular disorders) after an uneventful pregnancy in 39+3 weeks of gestation. His birth weight was 3060g, birth length 49cm, head circumference 34cm, APGAR 9/10, and no feeding difficulties or apneic episodes after birth were mentioned.

He presented distinct muscular hypotonia and congenital clubfoot on the right side as well as motoric developmental delay since birth. Proprioceptive reflexes were normal, as well as laboratory workup with normal creatine kinase. Motor milestones: at the age of 16 months, he was able to sit independently and started crawling. At the age of 24 months, he is able to stand with assistance, but is not able to stand or walk independently. Until 24 months he was not able to speak understandable words but uses syllables, his speech comprehension and behavior is supposed to be normal. The patient was last reviewed at the age of 24 months (height 87cm, SDS: -0.40 / $P_{34.6}$, weight 8800g SDS: -2.76 / $P_{0.3}$, BMI: 11.63kg/m^2 (SDS: -5.02 / $P_{0.0}$), head circumference: 46.0cm (SDS: -2.54 / $P_{0.6}$), dolichocephaly and signs of myopathic facial expression as well as a subtle sacral dimple were observed during physical examination. He is dystrophic and needs hypercaloric nutritional supplements.

Family 27: c.746T>C, p.(Met249Thr)
Proband P27.1 is male and the first child born to a 20-year-old mother. Pregnancy and full-term delivery were normal without exposures or complications. Birth weight was 3.685 kg and length was 56 cm. No neonatal complications were noted. His physical growth has been normal, following high percentiles for height and weight.

During his first year of life he underwent surgical correction of metopic and sagittal craniosynostosis at 5 months of age. He was diagnosed with a moderate combined obstructive and central sleep apnea with a mild oxygen desaturation. He was also found to have coughing and choking spells with periods of apnea and cyanosis during periods of wakefulness, but has since recovered. He was found to have thoracic syringohydromyelia.

He has global developmental delay including speech and both fine and gross motor. He began crawling at 16 months and walking at 3 years. He has mild generalized hypotonia and symmetrically hypoactive tendon reflexes. His hearing and vision appear to be normal. He has a history of heart murmur, but no definite evidence of congenital heart defects or heart involvement. He has trivial tricuspid valve regurgitation and possible left ventricular hypertrophy by echocardiogram at 1 year of age. Head MRI, EEG, CT of the chest, and swallow study were normal.

Physical exam revealed dysmorphic features including mild trigonocephaly with prominence of the vertex area with flattening of the occipitoparietal area, up-slanted palpebral fissures, wide-set eyes with epicanthal folds, mild bilateral ptosis, pseudostrabismus, mild synophrys, short upturned nose, and thick alveolar ridges. The palate is slightly high-arched, and he has micrognathia, a hypoplastic mandible, and prominent incisors. He has bitemporal narrowing even after craniosynostosis surgical correction. He has somewhat tapered digits. There was mild diffuse hypotonia. His facial features are similar to his mother and his sister. No skin abnormalities or pigmentary findings were noted. Normal male genitalia was observed. He has restless sleep and habitual snoring. He also has behavioral challenges including frequent temper tantrums, occasional aggressive behavior, ADHD, anxiety, depression, and poor social skills.

The sister (P27.2) of the proband is similarly affected and has global developmental delay, intellectual disability, esotropia, and obstructive and central sleep apnea, status post tonsillectomy and adenoidectomy, conductive hearing loss status post PE tube placement. Her physical development has been normal for height and weight. She has minimal axial hypotonia. She sat at 18 months of age, crawled at 19-20 months of age, and made her first steps at 22 months. She has relative macrocephaly, up-slanting palpebral fissures with epicanthal folds, and her nose is short and mouth is small and triangular. Her MRI showed white matter volume loss. She has difficulties with sleep and has significant anxiety.

The mother (P27.3) also has a history of developmental delay with fine and gross motor delays as well as speech delay, failing to talk until 7 years of age and having speech apraxia, ADHD, depression, anxiety disorder, hypothyroidism, macrocephaly, and restless leg syndrome. A genetic evaluation at an outside institution at 2 years of age reportedly did not disclose an etiology for her features. The father has history of ADHD, depression, and learning difficulties. He graduated high school and completed an additional year of college with the aid of special education. Other relevant family history included a paternal half-sister, 11 years old, with ADHD, and a paternal half-brother who was reportedly stillborn at 8 months gestation.

Proband P27.1 had prior unrevealing genetic testing including karyotype, chromosomal microarray (Agilent 44K oligonucleotide array), *RAI1* sequencing, and Fragile X testing. Additional biochemical studies were also uninformative. Transferrin isoforms, creatine kinase, plasma ammonia, sensitive TSH, and screening for Smith-Lemli-Opitz syndrome were all within normal limits. Clinical exome sequencing by Baylor Genetics (Houston, TX) on P27.1 with targeted segregation testing by Sanger sequencing for the sister (P27.2), mother (P27.3), and the father identified the c.746T>C, p.(Met249Thr) VUS in *KCNK9* heterozygous in the all three similarly affected family members (P27.1, P27.2, and P27.3) (4). Further segregation studies in the maternal grandparents indicate the variant was likely *de novo* in the mother (P27.3) and raised the variant classification to likely pathogenic. Additional variants identified by exome sequencing include a paternal heterozygous VUS in *ERF* (NM_006494.3: c.1516G>A; p.(Asp506Asn)) may be contributing to the craniosynostosis observed in P27.1 and macrocephaly in his mother (P27.3), but further studies are needed. The sister (P27.2) was negative for this variant. A VUS in *ARID1A* (NM_006015.6:c.5812A>G; p.(Lys1938Glu)) was identified in proband (P27.1), sister

(P27.2), and mother (P27.3). A heterozygous VUS in *CDH15* (NM_004933.2: c.1232+1G>T; p.?) was also identified in proband (P27.1), sister (P27.2), and mother (P27.3).

Family 29: c.958G>A, p.(Ala320Thr)
Proband P29.1 is male and was born to a 19-year-old G2P2A0 mother at approximately 36 weeks gestation (exact gestational age not known) via spontaneous vaginal delivery without prenatal or neonatal complications. His birth weight was 5lb 11oz. Developmental delay was noted at approximately 6 months of age. He established care with the genetics clinic at age 19 months for evaluation of macrocephaly with a large open fontanelle and developmental delay. Chromosome microarray analysis (CMA, Baylor Genetics, CMA-HR+SNP V8.3) was completed with normal results. Follow-up in genetics clinic at age 4 years is notable for recurrent Staphylococcus skin infections in addition to unilateral hearing impairment, speech concerns, and aggressive behaviors. He had two febrile seizures at 2 and 5 years of age. Follow-up in genetics clinic at age 5 years 7 months noted concern about fine motor delay and ongoing concerns for speech delay. He was diagnosed with ADHD and mild cognitive delays through the school district. He also behavioral concerns including attention problems, anger, oppositional behaviors (talking back, not listening, throwing/hitting things when angry). Brain MRI revealed no brain lesion to specifically account for seizures. An updated version of CMA-HR+SNP (V10.2) was completed with normal results. Clinical exome sequencing (V3) at Baylor Genetics (Houston, TX) revealed the maternally inherited c.958G>A, p.(Ala320Thr) variant in *KCNK9*.

Molecular Modeling Reveals Mutation-Specific Effects on Channel Mechanics

We assessed folding stability changes ($\Delta\Delta G_{fold}$) and p.Gly236Arg was predicted to be highly stabilizing, ten variants (p.Arg131Ser, p.Arg131His, p.Arg131Pro, p.Met132Arg, p.Met156V, p.Met159Ile, p.Phe164Cys, p.Tyr205Cys, p.Ala237Asp, p.Met249Thr,) were predicted to be moderately to highly destabilizing, and two variants (p.Thr199Ala p.Ala237Thr) were not predicted to significantly affect stability (**Table 2**). The double-mutation of Gly236Arg+Ala237Thr did not significantly resolve the change in folding free energy ($\Delta\Delta G_{fold}$) but did resolve some of the differences between Gly236Arg and WT, such as 4-body potential energy score (which accounts for shape and solvation). Stabilizing variants such as Gly236Arg will lock the protein in one conformation and impair its ability to transport ions, compared to destabilizing variants which may lead to a misfolded protein, or bias the channel away from normal productive conformations.

We also demonstrate specific mechanistic differences among Arg131His/Ser/Pro. While all three are predicted to be dysregulated in their dynamics, the way they are dysregulated differs. Arg131His exhibited greater destabilization of the selectivity filter residues, compared to other Arg131 variants, both in the distortion of its shape (RMSD) and by increased mobilities (RMSF). Arg131Ser, while less distorted in its selectivity filter overall, also had more WT-like alterations to the TIG motif sidechains, compared to other Arg131 variants, although display the most alteration to the 3-HBs. Arg131Pro exhibited antechamber opening by PCA, while the other two exhibited moderate closing. Thus, dysregulation of *KCNK9* by pathogenic genetic variation likely induce different effects on the TASK3 protein, even when occurring at the same position

The direction of conformational shift may be less important than magnitude. Thus, we used principal component (PC) analysis to quantify conformational shifts across our full dataset (**Additional file 6: Fig. S1**). The first three PCs (see **Additional file 3: Video S2** for visualization) account for collective motions that couple changes at the cytoplasmic and extracellular sides of

the channel, including gating (PC1), opening of the intracellular side of the channel (PC2), and interior antechamber expansion (PC3). We summarized how each variant leads to changes in protein motions, quantified by their different average positions along these three PCs (**Table 2**). We manually inspected the PCs and annotated which directions of each PC corresponded to, for example, channel gating. Certain variants, including Gly236Arg and Arg131Ser, were associated with large shifts in multiple PCs, that collectively indicated a more closed state at the intracellular-facing side. Other variants occurring at the same sites (e.g., Arg131Pro and Arg131His), however, were associated only with modest changes in conformation. Thus, protein simulations were able to provide information about which genetic variants were more likely to alter conformation of the channel. We found the dynamics data informative for interpreting a change in channel function for 11/14 variants. Together, these calculations inform on both the dysfunctional state induced by each variant while at the same time provides valuable inferences on the likely mechanisms underlying their defects.

Molecular Dynamics Simulations Show Changes in Potassium Ion Distribution

To assess how variants may affect ion transport, we further quantified changes to the selectivity filter, which is characterized by a ring of hydrophobic and aromatic residues that form interactions between monomers and facilitate transport through the channel (**Additional file 6: Fig. S2B**). Dynamic changes lead to rearrangements of the selectivity filter, as observed for Arg131His and Phe135del (**Table 2**), which may disrupt channel function. We quantified changes to K+ distribution at key locations (**Additional file 6: Fig. S3 and S4**) and found severe depletion of K^+ nearby the selectivity filter for Gly236Arg (**Fig. 3A**). At the cytoplasmic face, Gly236Arg showed nearly no K+, while Arg131His showed little, and Met159Ile comparatively more but still diminished compared to WT (**Fig. 3B**). Previous biochemical studies identified a partially compensating variant, Ala237Thr, for Gly236Arg; the double-mutant K+ distributions were more WT-like at multiple sites (**Additional file 6: Fig. S3 and S4**) including at the selectivity filter (**Fig. 3C**) (5, 6). We observed that the PC2 shift for Gly236Arg did not occur for the double-mutant. Instead, PC1 and PC3 shifted, changing the shape of the central chamber and likely altering access through the intracellular-facing side. Interestingly, $[K^+]$ around the selectivity filter and $\Delta\Delta G_{fold}$ were significantly correlated ($\rho = 0.50$), indicating a link between our structure and dynamics based calculations. Thus, 3D structure-based scores were indicative of changes to K+ distributions around TASK3, but simulations provided greater sensitivity. The simulation length was sufficient to observe multiple ion transport events, which can support predictions of conductance. We observed more (>2) ion transport events than WT (2) in most simulations, especially for Met132Arg (14), Thr199Ala (12), and Arg131Pro (10), and fewer for Gly236Arg (0) and Met249Thr (1). Thus, 3D structural and time-dynamic data enriched the information available for explaining the effect of each variant on channel function.

References:

1. Aspromonte MC, Bellini M, Gasparini A, Carraro M, Bettella E, Polli R, et al. Characterization of intellectual disability and autism comorbidity through gene panel sequencing. Hum Mutat. 2019;40(9):1346-63.

2. Larcher L, Norris JW, Lejeune E, Buratti J, Mignot C, Garel C, et al. The complete loss of function of the SMS gene results in a severe form of Snyder-Robinson syndrome. Eur J Med Genet. 2020;63(4):103777.

3. Graham JM, Jr., Zadeh N, Kelley M, Tan ES, Liew W, Tan V, et al. KCNK9 imprinting syndrome-further delineation of a possible treatable disorder. Am J Med Genet A. 2016;170(10):2632-7.

4. Klee EW, Cousin MA, Pinto EVF, Morales-Rosado JA, Macke EL, Jenkinson WG, et al. Impact of integrated translational research on clinical exome sequencing. Genet Med. 2021;23(3):498-507.

5. Ashmole I, Vavoulis DV, Stansfeld PJ, Mehta PR, Feng JF, Sutcliffe MJ, et al. The response of the tandem pore potassium channel TASK-3 (K(2P)9.1) to voltage: gating at the cytoplasmic mouth. J Physiol. 2009;587(Pt 20):4769-83.

6. Veale EL, Hassan M, Walsh Y, Al-Moubarak E, Mathie A. Recovery of current through mutated TASK3 potassium channels underlying Birk Barel syndrome. Mol Pharmacol. 2014;85(3):397-407.
