# Supplementary material for: Gain and loss of TASK3 channel function and its regulation by novel variation cause KCNK9 imprinting syndrome
Source: Genome Med. 2022 Jun 13;14:62. doi: 10.1186/s13073-022-01064-4 (PMC9195326; doi:10.1186/s13073-022-01064-4)
Supplement: Supplementary file 5 — Additional file 5: Table S2. Seizure and seizure-like episodes in affected individuals with at least one afebrile seizure. [file 13073_2022_1064_MOESM5_ESM.pdf]

Gain and loss of TASK3 channel function and its regulation by novel variation cause *KCNK9* imprinting syndrome

Additional file 5

Table S2. Seizure and seizure-like episodes in affected individuals with at least one afebrile seizure. y = years, m = months, d = days, EEG = electroencephalogram

Identifier	Age at febrile episode	Age at afebrile episode	Episode description	EEG findings	Treatment
P11.1	3.5y		Status epilepticus		
		8y	Tonic seizures	Spike and wave discharges	well controlled by lamotrigine
P12.1		2y2m	Seizures		sodium valproate, lamotrigine
P13.1		42d	Myoclonic spasms	Intermittent pseudo periodic pattern and no epileptic discharges	
		2y4m	Brief episodes of tonic limb flexion associated with gaze fixation		
		3y	Status epilepticus	Compatible with Lennox-Gastaut	Well controlled by valproate, lamotrigine, clonazepam
P18.1		1m	Episode concerning for seizure	No epileptiform activity	
		1y3m	Episode concerning for seizure	No epileptiform activity	
		5y11m	Focal seizures	Multifocal spike discharges with atypical generalized spike waves and bitemporal slowing, and generalized paroxysmal fast activity with maximal impact at right temporo-parieto-occipital area	Well controlled by oxcarbazepine
F22 (5/15 individuals)	no information				
