# Supplementary material for: Gain and loss of TASK3 channel function and its regulation by novel variation cause KCNK9 imprinting syndrome
Source: Genome Med. 2022 Jun 13;14:62. doi: 10.1186/s13073-022-01064-4 (PMC9195326; doi:10.1186/s13073-022-01064-4)
Supplement: Supplementary file 6 — Additional file 6: Figure S1. TASK3 conformational changes define PC motions and channel gating. Figure S2. Detailed view of the TASK3 selectivity filter and surrounding residues. Figure S3. Distributions of K+ ions for selected positions along the transport process, and across variants. Figure S4. Genomic variants lead to changes in K+ concentration at the selectivity filter. Figure S5. Cellular localization of labelled TASK3 variants. Figure S6. Comparison of whole cell current density for Tyr205Cys in the presence of various cysteine-modifying agents. [file 13073_2022_1064_MOESM6_ESM.pdf]

Gain and loss of TASK3 channel function and its regulation by novel variation cause *KCNK9* imprinting syndrome

Additional file 6

Figures S1-S6

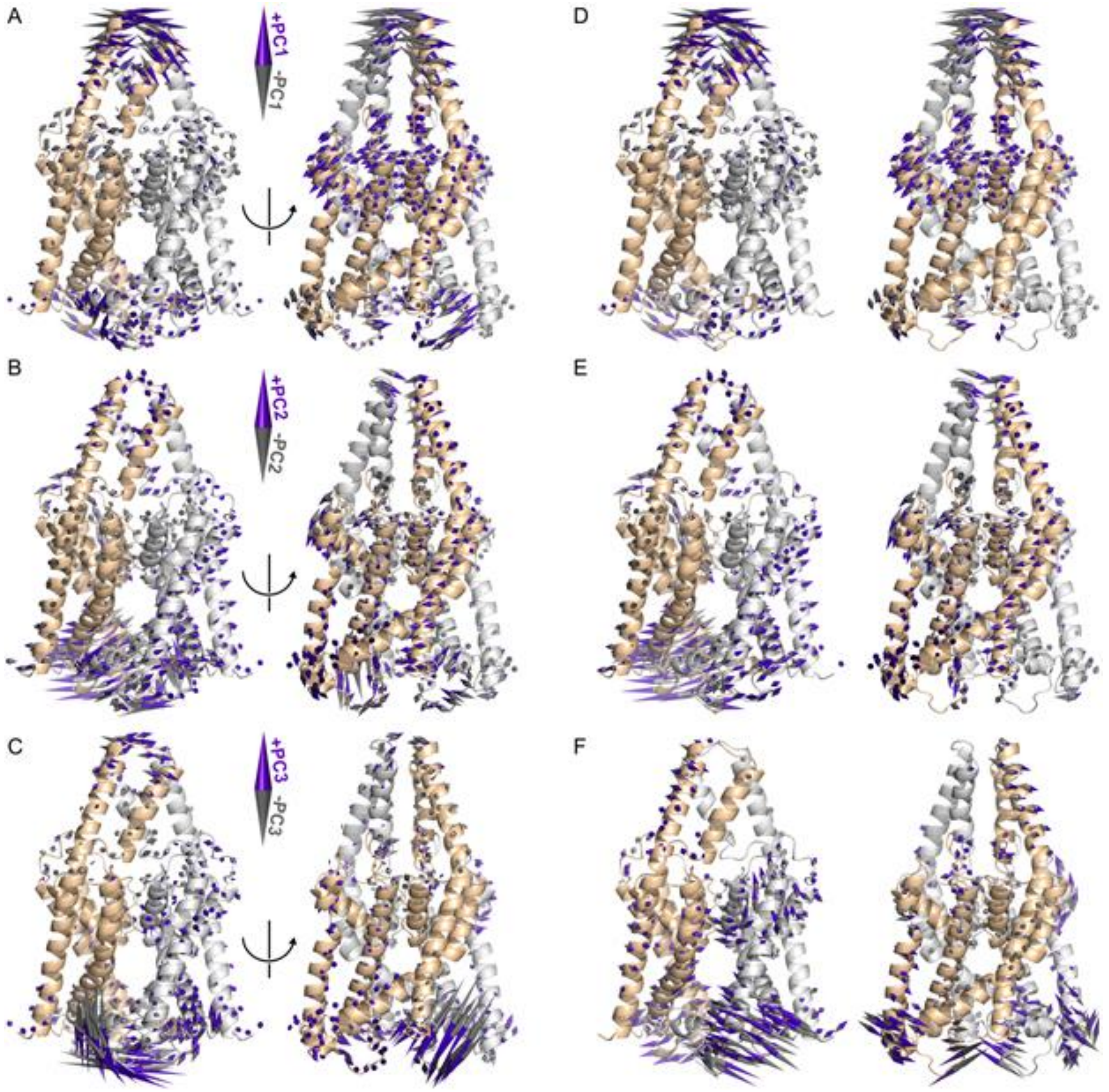

Figure S1: TASK3 conformational changes define PC motions and channel gating.
We used PC analysis to summarize the most predominant motions across all simulations. First, we *excluded the C-terminus*, to analyze patterns elsewhere in the protein. The direction and relative magnitude of PC motion is indicated using cones centered on each amino acid. **A** PC1 corresponds to an opposing motion between the extracellular helices and the cytoplasmic side of the channel. **B** PC2 primarily corresponds to motion of the outer loops of one monomer outwards, while the trans-membrane helices of the other monomer also move outwards. Thus, activation of +PC2 will generate a more open conformation at the cytoplasmic face as well as a greater interior volume. **C** PC3 corresponds to a motion that closely resembles PC2, but with the activity between the two monomers switched. We next show the analogues visualizations *when the C-terminus is included*. **D** PC1 corresponds to an opposing motion between the extracellular helices and the top of the channel. The cytoplasmic side of each helix moves in the opposite direction as the extracellular side of the same helix. **E** PC2 corresponds to movement of the C-terminal helices into a position in front of the channel opening (+PC2) or upwards and to the outside of the protein (-PC2). **F** PC3 moves the C-terminus along the membrane plane, with coupled motion of one monomer's C-terminal helix perpendicular to the membrane plane.

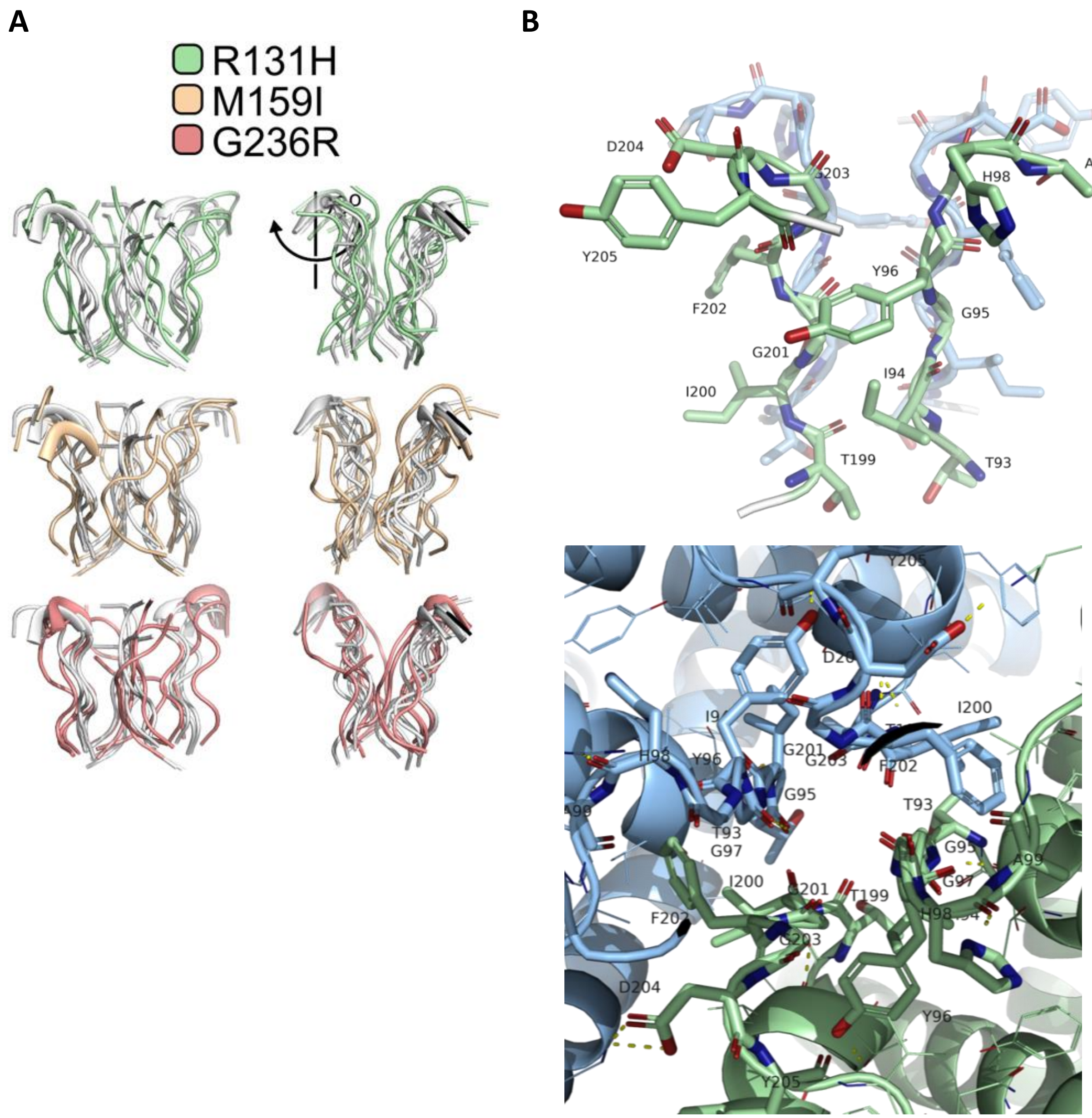

Figure S2: Detailed view of the TASK3 selectivity filter and surrounding residues.
A We show three example variants' selectivity filters from the end of simulations. Triplicates of WT are in white and of each variant colored distinctly. There is greater conformational variability at the selectivity filter for proteins encoded by these genetic variants (quantified in **Table S1**). **B, upper** Viewed from a similar angle, we show the selectivity filter residues with one monomer colored green and the other blue. **B, lower** Viewed from the extracellular side of the initial WT conformation. Hydrogen bonds are represented by yellow dashed lines, residues directly making up the selectivity filter are shown as sticks, and those within 2.7Å as lines.

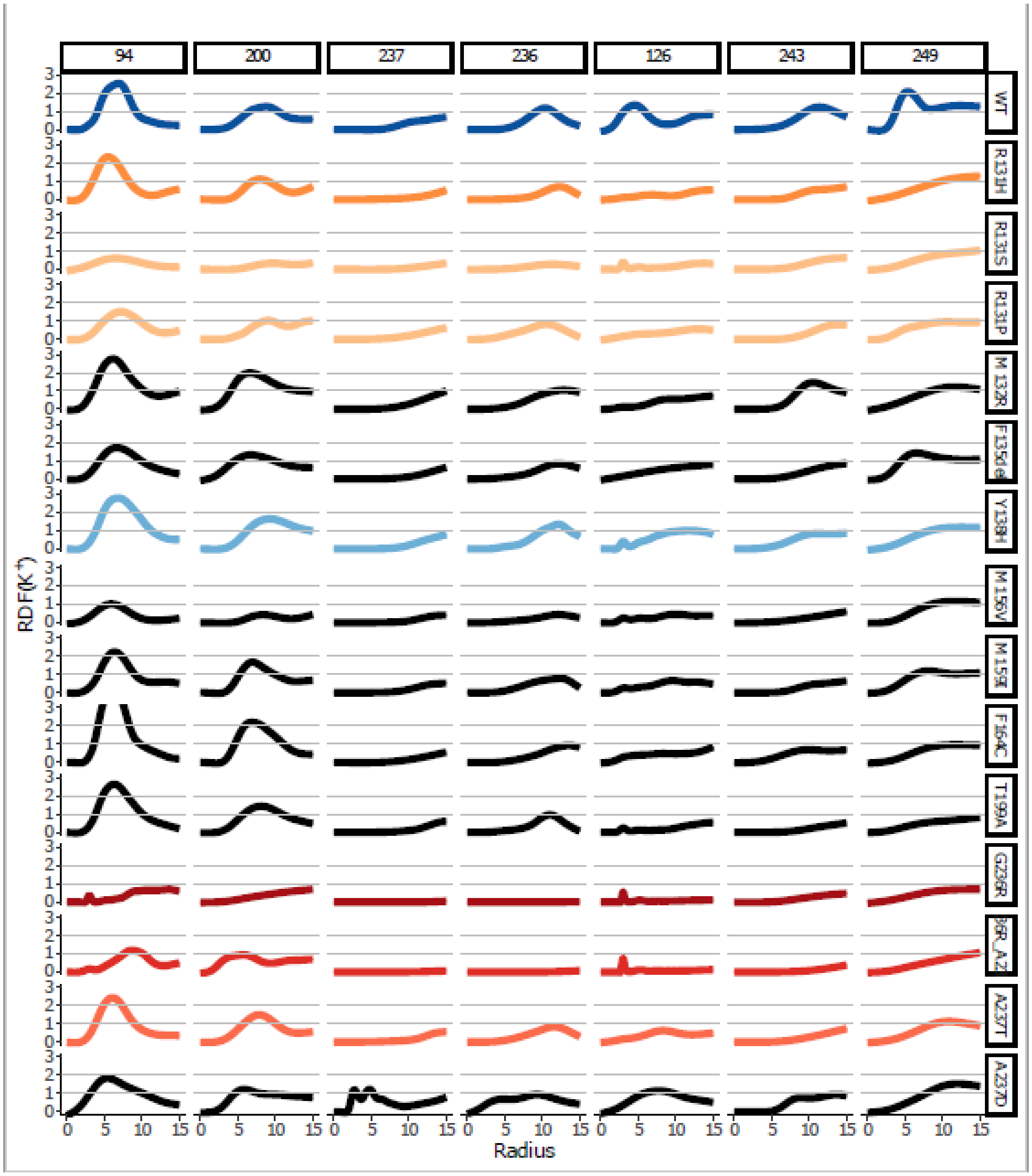

Figure S3: Distributions of K^+ ions for selected positions along the transport process, and across variants.

We plot the average K^+ RDF centered at each indicated residue position - each column is the RDF centered on the residue index in the column label. Color is by the nature of each variant: blues, expected benign; yellow, rescue-mutation pair; orange, VUS or pathogenic but with atypical presentation; red, well-characterized pathogenic mutation.

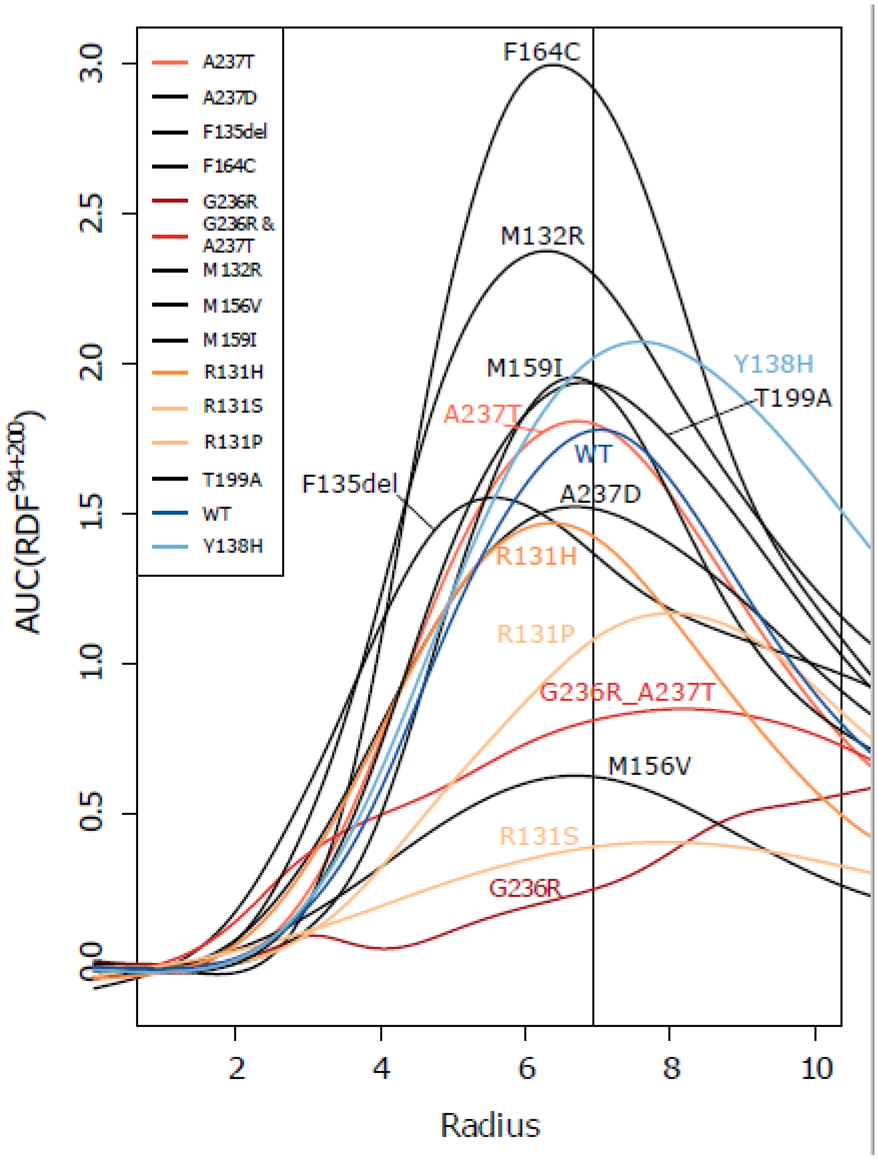

Figure S4: Genomic variants lead to changes in K^+ concentration at the selectivity filter. We show the RDF of K^+ around the base of the selectivity filter, defined by the geometric center of residues 94 and 200 from both monomers, for each variant. The distribution was measured across time and replicates of MD simulations and a spline fit applied for visualization.

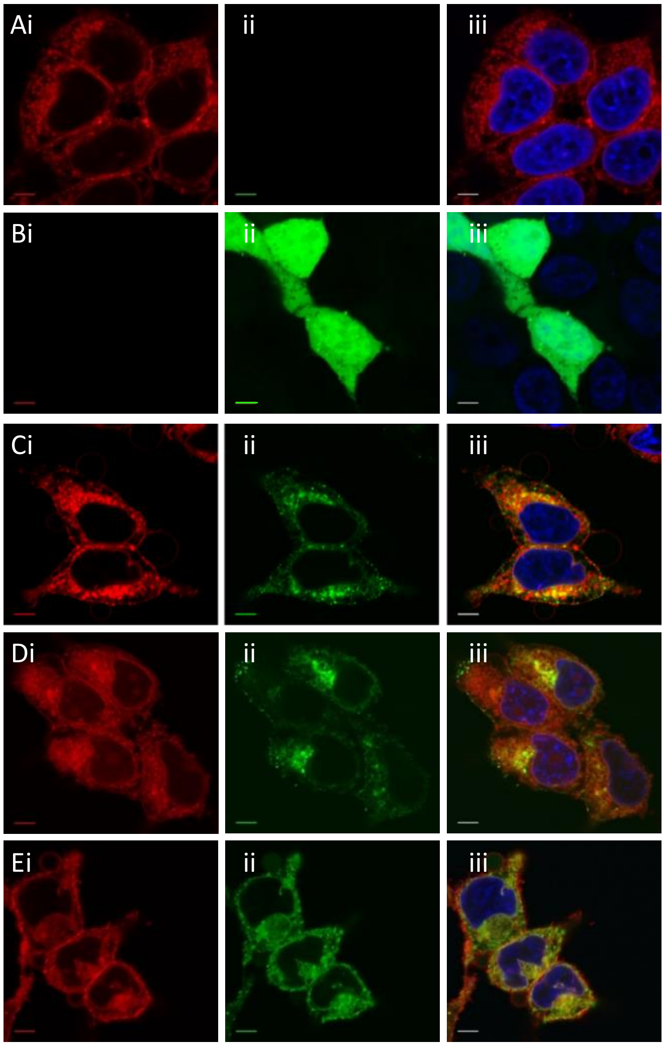

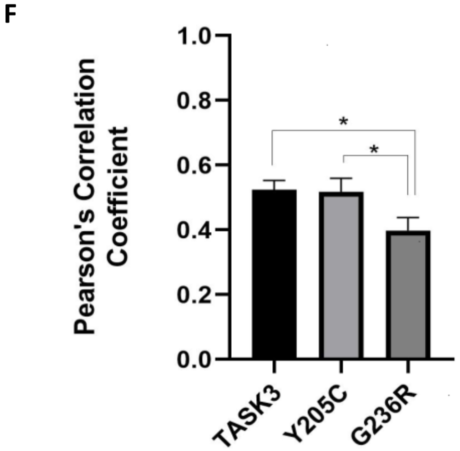

Figure S5: Cellular localization of labelled TASK3 variants.

Confocal images **Ai-iii** represent a single-stain control image consisting of membrane stained, untransfected tsA201 cells. [Ai] Displays the red stained plasma membrane; Aii no green autofluorescence and [Aiii] is the overlap of [Ai] and [Aii]. Nuclei are blue. **Bi-iii** A single-stain control image of tsA201 cells transfected with GFP only. **Ci-iii)** As above for transiently transfected WT-TASK3_GFP with overlay of images [Ci] and [Cii] in [Ciii] resulting in a yellow signal indicative of channel colocalisation at the plasma membrane. **Di-Diii** as above for TASK3_G236R_GFP; **Ei-iii** as above for TASK3_Y205C_GFP mutation. All scale bars are 5 µM. **F** A histogram comparing PCC quantification of colocalization of images [C-F]. Error bars represent standard error of the mean (SEM).

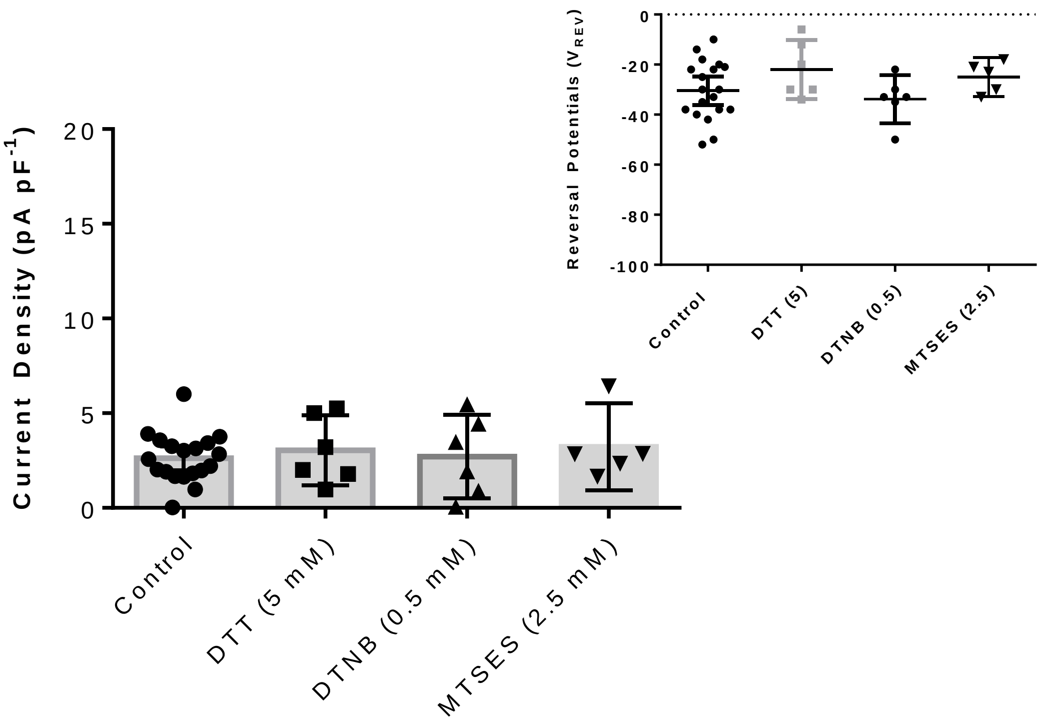

Figure S6: Comparison of whole cell current density for Tyr205Cys in the presence of various cysteine-modifying agents.

Graph of current density (pA pF^{-1}) measured from cells transiently expressing Tyr205Cys channels in a physiological extracellular solution (control) of 2.5 mM [K^+] either alone or including 5 mM DTT, 0.5 mM DTNB or 2.5 mM *MTSES (Ellman's reagent)*. The average whole-cell current measured as a difference between current seen at −40 mV and −80 mV remained unchanged ($p > 0.05$, One-way ANOVA) in all experimental conditions: 2.6 pA pF^{-1} [95% Confidence Intervals (CI): 2.0 to 3.2, n = 19] for control; 3.0 pA pF−1 [95% CI: 1.2 to 4.9, n = 6] for DTT; 2.7 pA pF−1 [95% CI: 0.5 to 4.9, n = 6] for DTNB; and 3.2 pA pF^{-1} [95% CI: 0.9 to 5.5, n = 5] for MTSES. Similarly, no change ($p > 0.05$) was observed in the mean zero current reversal potential (V_{REV}) of cells recorded in each experimental condition (Inset): −30 mV [95% CI: −36 to −25, n = 19] for control; −22 mV [95% CI: −34 to −10, n = 6] for DTT; −34 mV [95% CI: −43 to −24, n = 6] for DTNB; and −25 mV [95% CI: −36 to −17, n = 5] for MTSES. Abbreviations: DTT – dithiothreitol; DTNB - Dithio-bis(2-nitrobenzoic acid; MTSES - Sodium (2-Sulfonatoethyl) Methanethiosulfonate; mM – milimolar, mV – millivolt, pA – picoamp, pF – picofarad. Symbols represent the number of cells recorded per condition.
