# Supplementary material for: Gain and loss of TASK3 channel function and its regulation by novel variation cause KCNK9 imprinting syndrome
Source: Genome Med. 2022 Jun 13;14:62. doi: 10.1186/s13073-022-01064-4 (PMC9195326; doi:10.1186/s13073-022-01064-4)
Supplement: Supplementary file 7 — Additional file 7: Table S3. A comparison of whole cell current density and reversal potentials between TASK3 clinical variants and matched WT controls. [file 13073_2022_1064_MOESM7_ESM.pdf]

Gain and loss of TASK3 channel function and its regulation by novel variation cause *KCNK9* imprinting syndrome

Additional file 7

Table S3: A comparison of whole cell current density and reversal potentials between TASK3 clinical variants and matched WT controls. Current density (pA pF^{-1}) is measured as a difference of current (pA) seen at -40 mV and -80 mV, corrected for capacitance (pF). Error bars represent the 95% Confidence Intervals [CI] and the symbols represent individual data points. Current-voltage graphs were obtained from the averaged ramp changes in voltage between +20 and -120 mV for each variant and reversal potentials are expressed as the mean zero current level (mV) ± [95% CI]. n represents the number of individual cells (box inset).

Clinical Variant	Current Density (pA pF^{-1})	Current Voltage Relationship and reversal potentials (V_{REV})
TASK3_G236R	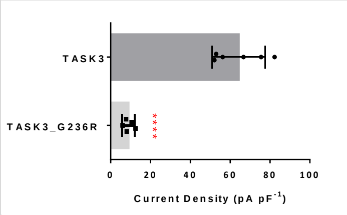	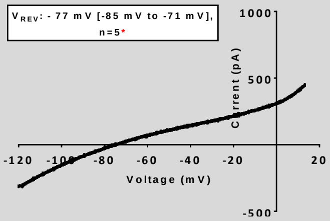
TASK3_R131C	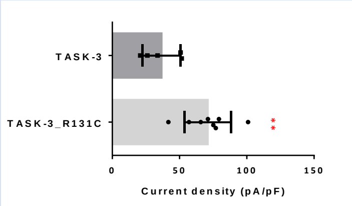	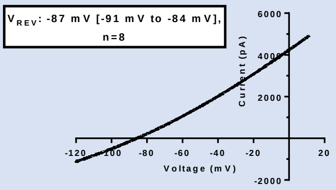
TASK3_R131H	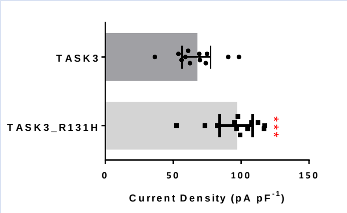	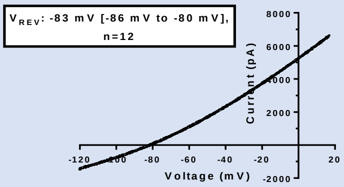
TASK3_R131P	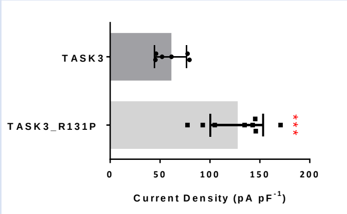	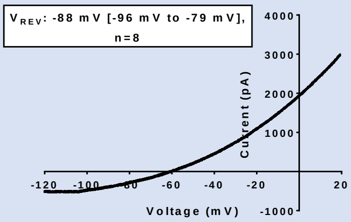

<table>
<tr><td>TASK3_R131S</td><td>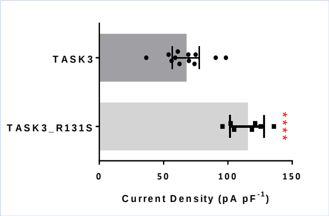</td><td>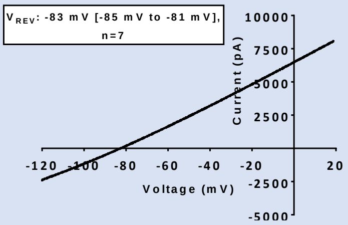</td></tr>
<tr><td>TASK3_M132R</td><td>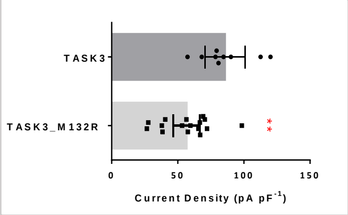</td><td>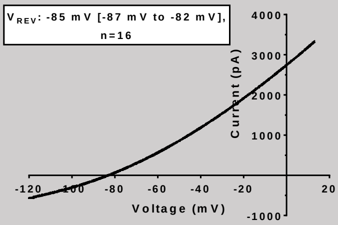</td></tr>
<tr><td>TASK3_F135deletion</td><td>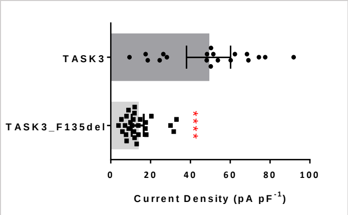</td><td>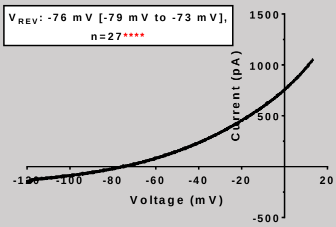</td></tr>
<tr><td>TASK3_M156V</td><td>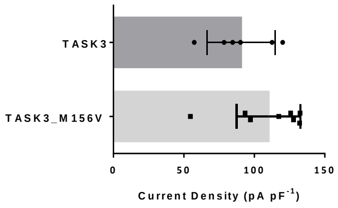</td><td>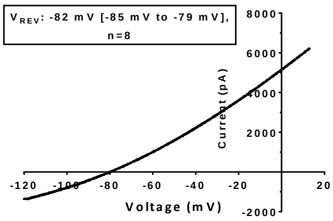</td></tr>
<tr><td>TASK3_M159I</td><td>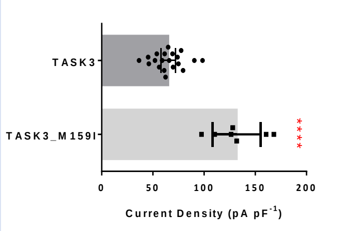</td><td>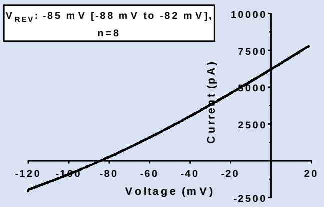</td></tr>
<tr><td>TASK3_F164C</td><td>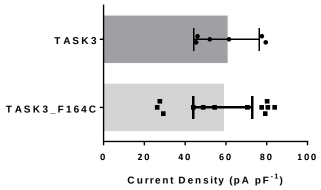</td><td>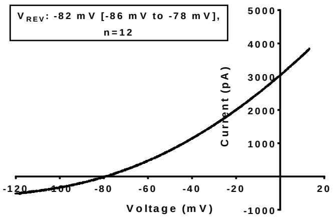</td></tr>
<tr><td>TASK3_T199A</td><td>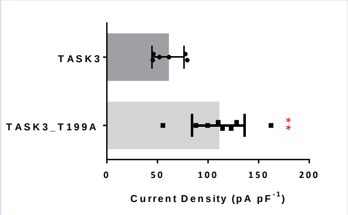</td><td>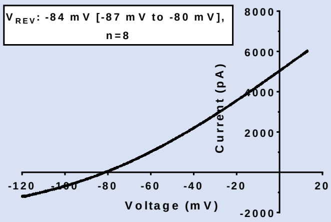</td></tr>
</table>

**TASK3_Y205C**	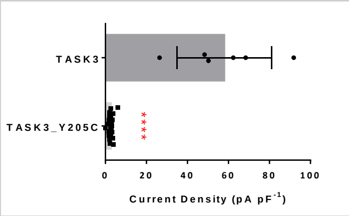	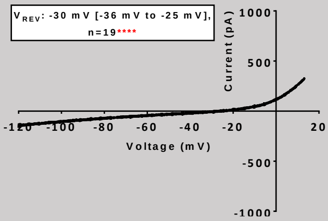
**TASK3_A237D**	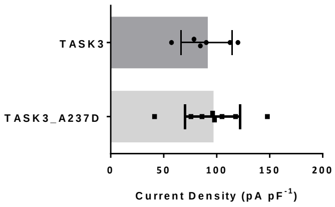	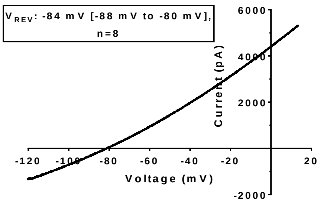
**TASK3_M249T**	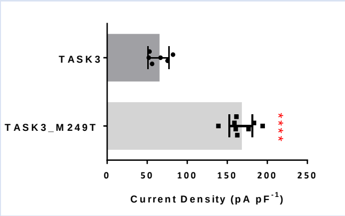	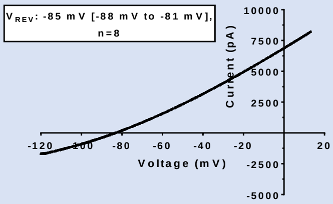
**TASK3_A320T**	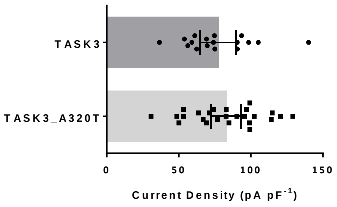	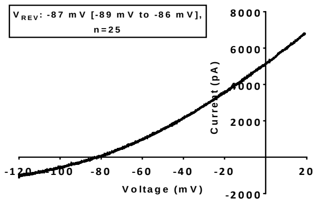

*p<0.05, **p<0.01, ***p<0.001 and ****p<0.0001 for between group differences determined using an unpaired Student's t-test. Boxes highlighted light blue represent a significant increase in current density, whilst grey highlighted boxes represent a significant decrease in current density and white highlighted boxes signify no recorded change in current density from WT. Abbreviations: mV – millivolt, pA – picoamp, pF – picofarad, V_{REV} – reversal potential.
