# Supplementary material for: Gain and loss of TASK3 channel function and its regulation by novel variation cause KCNK9 imprinting syndrome
Source: Genome Med. 2022 Jun 13;14:62. doi: 10.1186/s13073-022-01064-4 (PMC9195326; doi:10.1186/s13073-022-01064-4)
Supplement: Supplementary file 8 — Additional file 8: Table S4. A comparison of inhibition by extracellular acidification (pH 6.4) between TASK3 clinical variants and WT controls. [file 13073_2022_1064_MOESM8_ESM.pdf]

Gain and loss of TASK3 channel function and its regulation by novel variation cause *KCNK9* imprinting syndrome

Additional file 8

Table S4: A comparison of inhibition by extracellular acidification (pH 6.4) between TASK3 clinical variants and WT controls. Inhibition of current by pH 6.4 is calculated as the difference of current measured at pH 7.4, with that measured after exposure to pH 6.4, expressed as a percentage (%), displayed as a Box and Whiskers plot. Bars represent the min and max inhibition and lines the median inhibition, for each channel type. Points represent the individual data points. Sensitivity to extracellular acidification is represented in an exemplar time course plot demonstrating the effect of changing from an extracellular solution at pH 7.4, to one at pH 6.4 (black line). Each point is a 5 second (s) average of the difference current between that at -40 mV and that at -80 mV.

Clinical Variant	% Inhibition by extracellular acidification (pH 6.4)	Representative time course for inhibition by pH 6.4.
TASK3_G236R	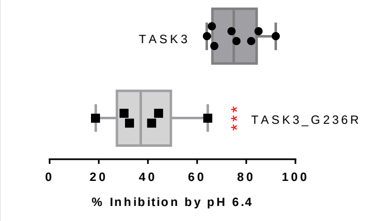	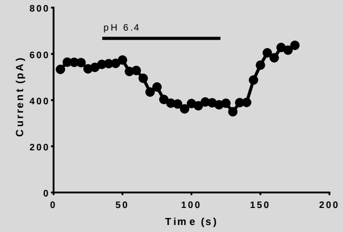
TASK3_R131C	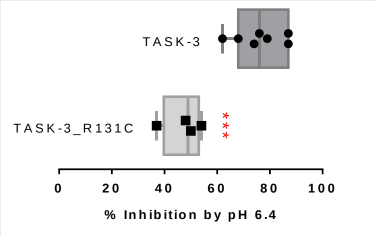	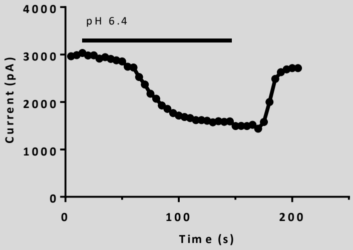
TASK3_R131H	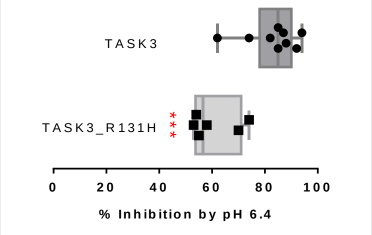	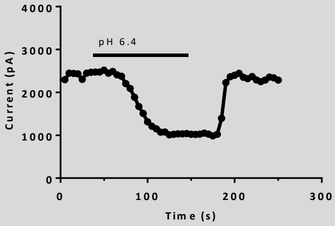

<table>
<tr><td>TASK3_R131P</td><td>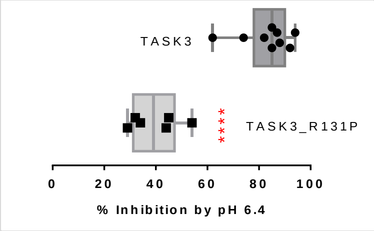</td><td>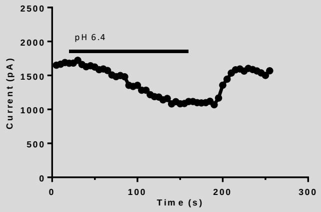</td></tr>
<tr><td>TASK3_R131S</td><td>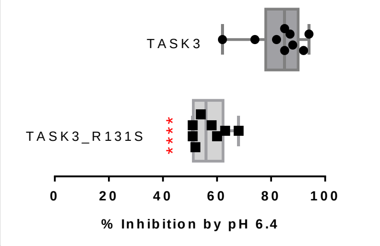</td><td>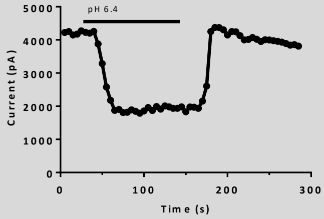</td></tr>
<tr><td>TASK3_M132R</td><td>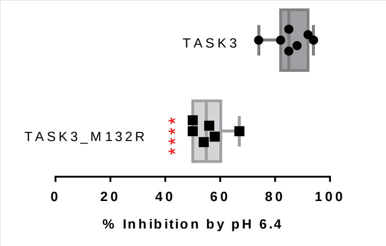</td><td>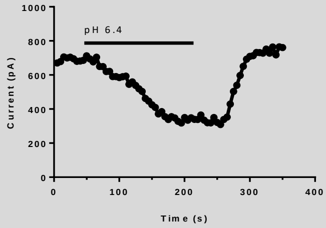</td></tr>
<tr><td>TASK3_F135deletion</td><td>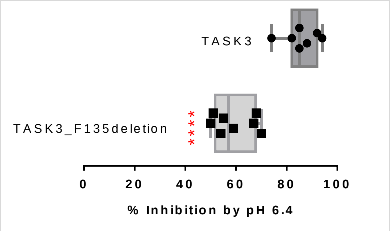</td><td>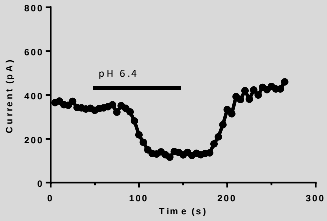</td></tr>
<tr><td>TASK3_M156V</td><td>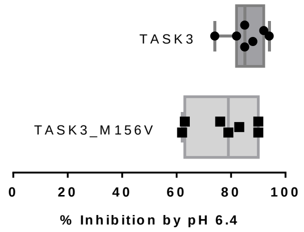</td><td>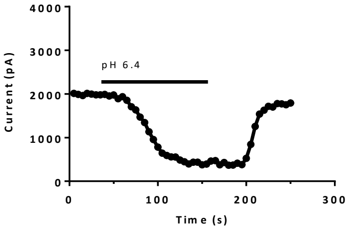</td></tr>
<tr><td>TASK3_M159I</td><td>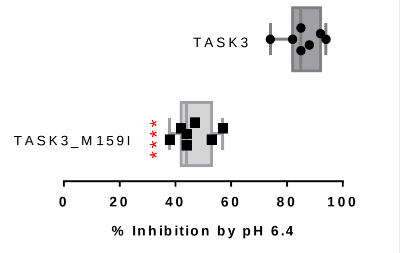</td><td>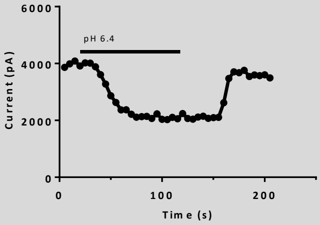</td></tr>
<tr><td>TASK3_F164C</td><td>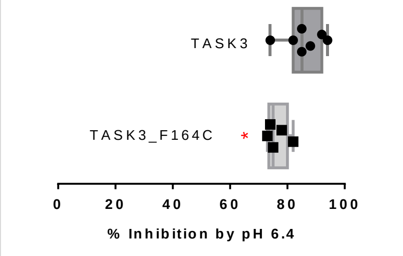</td><td>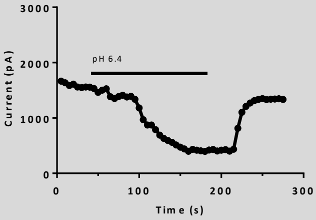</td></tr>
</table>

TASK3_T199A	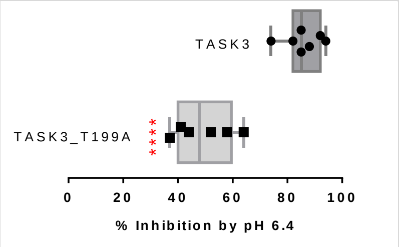	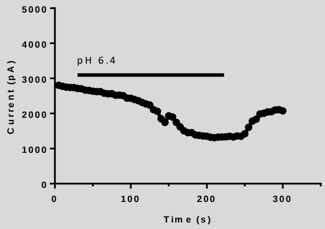
TASK3_A237D	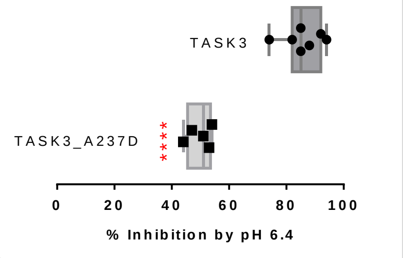	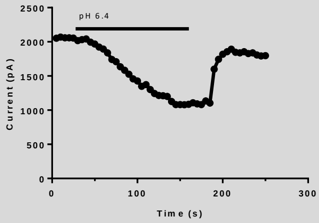
TASK3_M249T	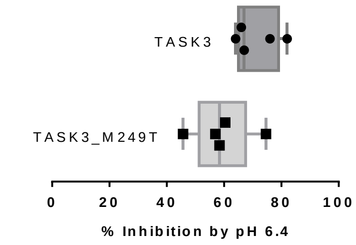	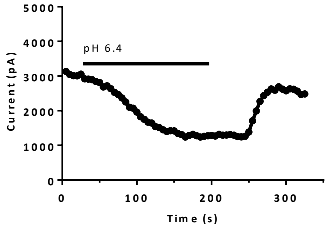
TASK3_A320T	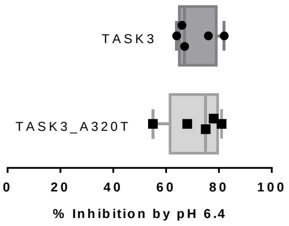	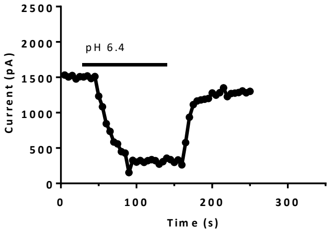

*p<0.05, **p<0.01, ***p<0.001 and ****p<0.0001 for between group differences determined using an unpaired Student's t-test. Boxes highlighted light blue represent a significant increase in sensitivity to extracellular acidification, whilst grey highlighted boxes represent a significant decrease in sensitivity and white highlighted boxes signify no recorded change in sensitivity from WT.
