# Supplementary material for: Gain and loss of TASK3 channel function and its regulation by novel variation cause KCNK9 imprinting syndrome
Source: Genome Med. 2022 Jun 13;14:62. doi: 10.1186/s13073-022-01064-4 (PMC9195326; doi:10.1186/s13073-022-01064-4)
Supplement: Supplementary file 9 — Additional file 9: Table S5. A comparison of GPCRs regulation between TASK3 clinical variants and WT controls. [file 13073_2022_1064_MOESM9_ESM.pdf]

Gain and loss of TASK3 channel function and its regulation by novel variation cause *KCNK9* imprinting syndrome

Additional file 9

Table S5: A comparison of GPCRs regulation between TASK3 clinical variants and WT controls. % inhibition is determined from the difference of current measured at in control solution, with that measured in the presence of the agonist, muscarine (0.1 µM), displayed as a Box and Whiskers plot. Bars represent the min and max inhibition and lines the median inhibition, for each channel type. Points represent the individual data points. Sensitivity to the acute application of the muscarinic receptor agonist, muscarine (0.1 µM) is represented in a time course plot for each variant demonstrating the inhibitory effect of applying muscarine (0.1 µM) (black line). Each point is a 5 s average of the difference current between that at -40 mV and that at -80 mV.

Clinical Variant	% Inhibition by muscarine (0.1 µM).	Representative time course demonstrating inhibition by muscarine (0.1 µM)
TASK3_G236R	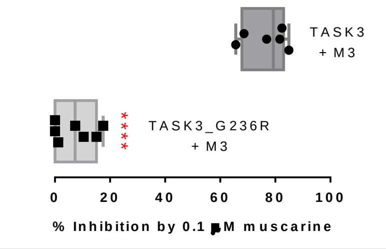	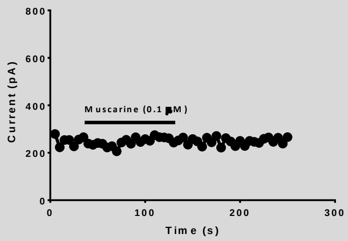
TASK3_R131C	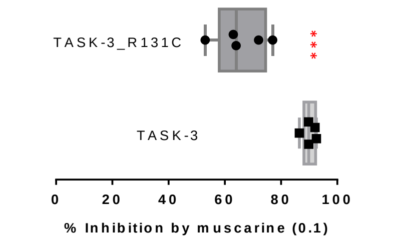	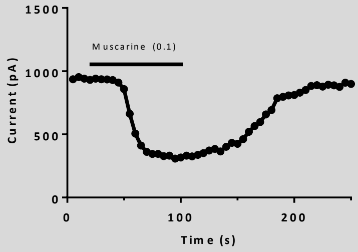
TASK3_R131H	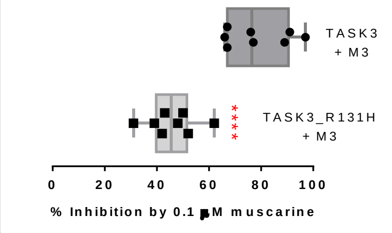	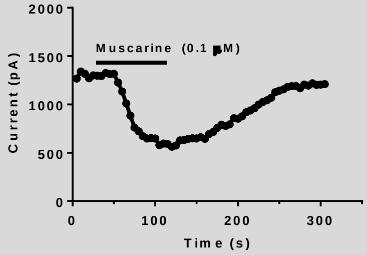

TASK3_R131P

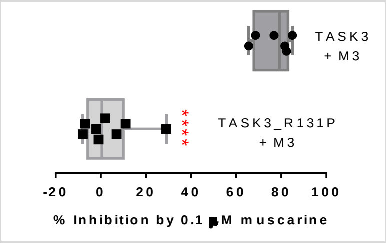

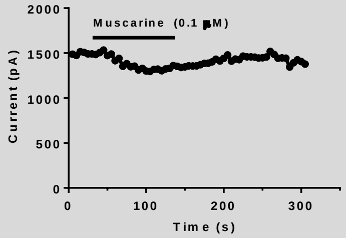

TASK3_R131S

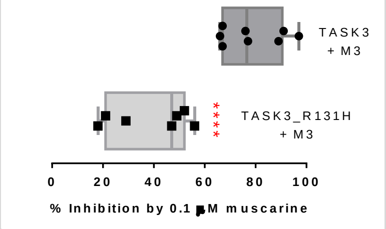

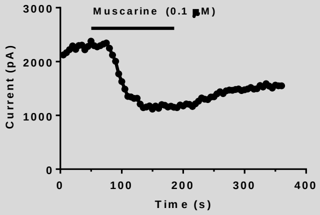

TASK3_M132R

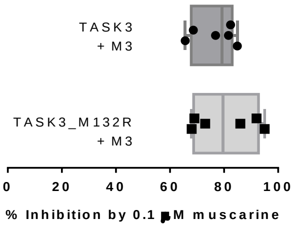

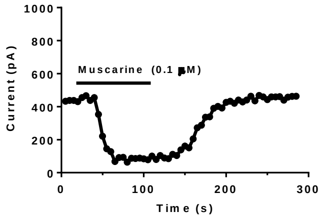

TASK3_F135deletion

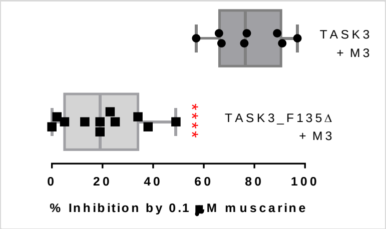

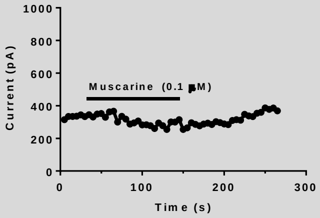

TASK3_M156V

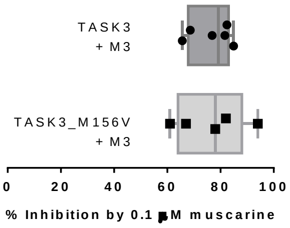

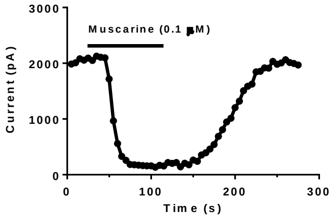

TASK3_M159I

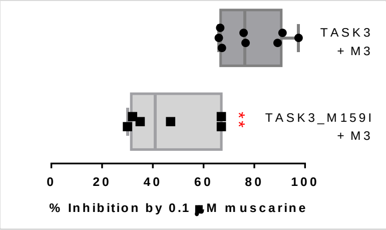

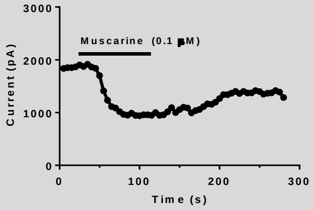

TASK3_F164C

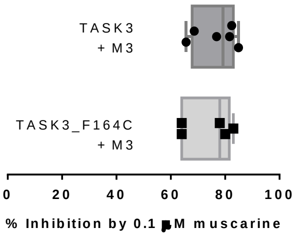

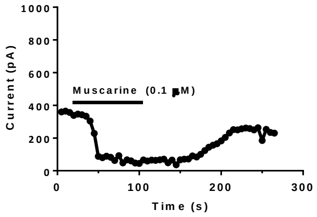

TASK3_T199A

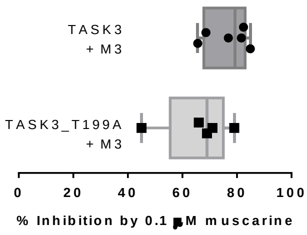

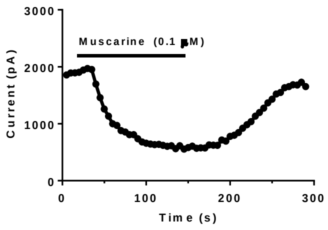

TASK3_A237D

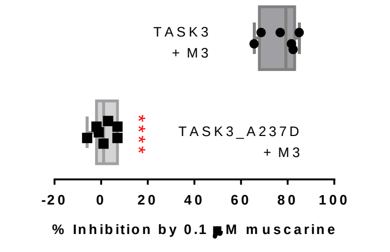

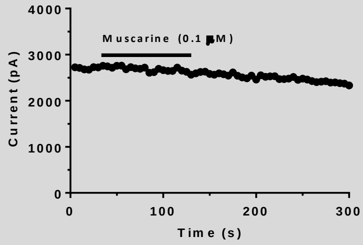

TASK3_M249T

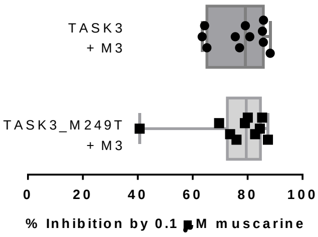

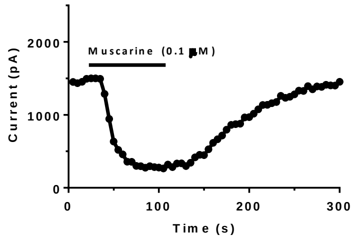

TASK3_A320T

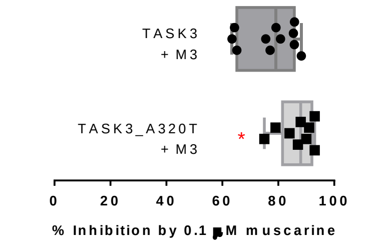

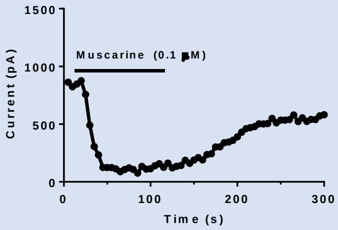

*p<0.05, **p<0.01 and ****p<0.0001 for between group differences determined using an unpaired Student's t-test. Boxes highlighted light blue represent a significant increase in sensitivity to GPCR regulation, whilst grey highlighted boxes represent a significant decrease in sensitivity and white highlighted boxes signify no change in sensitivity from WT.
